# Fighting *Pseudomonas aeruginosa* Infections: Antibacterial and Antibiofilm Activity of D-Q53 CecB, a Synthetic Analog of a Silkworm Natural Cecropin B Variant

**DOI:** 10.3390/ijms241512496

**Published:** 2023-08-06

**Authors:** Irene Varponi, Stefania Ferro, Luca Menilli, Alessandro Grapputo, Francesca Moret, Francesca Mastrotto, Oriano Marin, Federica Sandrelli

**Affiliations:** 1 Department of Biology, University of Padova, Via U. Bassi 58/B, 35131 Padova, Italy; irene.varponi@unipd.it (I.V.); luca.menilli@unipd.it (L.M.); alessandro.grapputo@unipd.it (A.G.); francesca.moret@unipd.it (F.M.); 2 Department of Biomedical Sciences, University of Padova, Via U. Bassi 58/B, 35131 Padova, Italy; stefania.ferro.1@unipd.it (S.F.); oriano.marin@unipd.it (O.M.); 3 National Biodiversity Future Centre, Piazza Marina 61, 90133 Palermo, Italy; 4 Department of Pharmaceutical and Pharmacological Sciences, University of Padova, Via F. Marzolo 5, 35131 Padova, Italy; francesca.mastrotto@unipd.it

**Keywords:** *Pseudomonas aeruginosa*, biofilm, antimicrobial activity, cecropin, AMP, protease degradation, enantiomer

## Abstract

*Pseudomonas aeruginosa* is an opportunistic Gram-negative bacterium responsible for severe nosocomial infections and is considered a critical pulmonary pathogen for both immunocompromised and cystic fibrosis patients. Planktonic cells of *P. aeruginosa* possess intrinsic and acquired resistances, inactivating several classes of conventional antibiotics. Additionally, this bacterium can grow, forming biofilms, and complex structures, further hampering the action of multiple antibiotics. Here, we report the biological properties of D-Q53 CecB, an all-D enantiomer of the silkworm natural peptide Q53 CecB. Compared to the L-variant, D-Q53 CecB was resistant to in vitro degradation by humans and *P. aeruginosa* elastases and showed an enhanced bactericidal activity against *P. aeruginosa* planktonic bacteria. D-Q53 CecB was thermostable and maintained its antimicrobial activity at high salt concentrations and in the presence of divalent cations or fetal-bovine serum, although at reduced levels. Against different types of human cells, D-Q53 CecB showed cytotoxic phenomena at concentrations several folds higher compared to those active against *P. aeruginosa.* When L- and D-Q53 CecB were compared for their antibiofilm properties, both peptides were active in inhibiting biofilm formation. However, the D-enantiomer was extremely effective in inducing biofilm degradation, suggesting this peptide as a favorable candidate in an anti-*Pseudomonas* therapy.

## 1. Introduction

*Pseudomonas aeruginosa* is an opportunistic Gram-negative pathogen representing a world-wide health burden as it is responsible for 10% of all nosocomial infections and one of the possible causes of emerging community-acquired infections [1]. Furthermore, *P. aeruginosa* is one of the most important pulmonary pathogens in both immunocompromised patients and individuals affected by Cystic Fibrosis (CF), with around 80% of CF patients infected by this opportunistic bacterium by adulthood [2].

Treatments of *P. aeruginosa* infections are extremely challenging as this bacterium can have both intrinsic and acquired mechanisms of antibiotic resistance, such as β-lactams, aminoglycosides, or quinolones [1,3] and for its capability to grow to form biofilms, a trait of adaptive resistance, which further hampers the action of antibiotics [1,4].

Biofilms are microorganisms’ complex communities growing on both biotic and abiotic substrates capable of promoting the expression of specific phenotypes to survive in adverse environmental conditions. In biofilms, bacterial cells are embedded in a self-generated extracellular matrix (ECM), which gives structural support and retains nutrients. ECM is composed of extracellular polysaccharides (EPS), lipids, proteins, and extracellular DNA (eDNA) [5,6]. *P. aeruginosa* forms robust biofilms, representing a physical barrier that impedes antibiotics from effectively reaching bacterial cells. Additionally, *P. aeruginosa* biofilms contain persister cells, phenotypic variants of bacteria characterized by metabolic inactivity and slow/absent growth, traits that increase tolerance to antibiotics [7]. After the removal of therapeutics, these dormant cells can reactivate proliferation generating new biofilms, hence contributing to making *P. aeruginosa* infections difficult to eradicate [8,9].

*P. aeruginosa* biofilm development is modulated by quorum sensing and occurs through stages that are known as attachment, maturation, and dispersion. Attachment is characterized by the initially reversible and then irreversible adhesion of bacterial cells to a substrate. Maturation includes the formation and expansion of bacterial microcolonies. During maturation, bacteria become more resistant to environmental stressors like antibiotic treatments. Finally, dispersion is characterized by the detachment of either planktonic bacteria or biofilm portions which form novel biofilms in new niches [5,6].

Initial phases of *P. aeruginosa* biofilm formation are favored by specific bacterial motilities, including swimming and swarming, which represent the movement of single cells and the coordinated movement of multiple bacteria, respectively [10]. Additionally, *P. aeruginosa* cells move inside the growing biofilm via twitching, jerking motility important for cell-to-cell interactions during microcolony formation [11,12]. After initial attachment, proliferating *P. aeruginosa* bacteria produce EPS components, including Polysaccharide synthesis locus (Psl), Pellicle (Pel), and alginate. Psl and Pel are involved in both biofilm formation and maturation, while alginate, typically produced by mucoid strains, has a role during maturation [13]. Recent analyses indicate that eDNA detected in ECM is also involved in biofilm formation as it has been shown to promote cell-cell adhesion and aggregation to a surface [5,14].

Both eDNA and EPSs have been shown to play major roles in biofilm antibiotic resistance. In a mature biofilm, eDNA was found to increase tolerance to some antimicrobials peptides (AMPs) (i.e., polymyxin and colistin) and aminoglycosides (i.e., tobramycin and gentamicin) [15]. This phenomenon has been associated with the eDNA’s ability to promote the expression of specific genes able to modify the Lipopolysaccharide (LPS) composition in the outer membrane of planktonic bacteria when treated with both types of antimicrobials [15]. However, some indications suggest that the negatively charged eDNA might also exert a protective function by binding the positively charged aminoglycosides, thus limiting their action [16]. EPS matrix was shown to give physical protection to the biofilm. In particular, against aminoglycosides, Psl was found to exert a defense role in the initial phases of biofilm formation [17] and Pel in mature biofilms [18]. Additionally, alginate has been proven to be a resistant factor against different classes of antibiotics and to provide protection against the host immune response [6].

In 2017, the World Health Organization (WHO) included *P. aeruginosa* among the critical priority pathogens for which new antimicrobial compounds are urgently required due to its globally increasing antibiotic resistance [19]. AMPs are considered potential alternatives to treat bacterial infections [20,21,22]. AMPs are a wide group of peptides produced by all organisms as the main effectors of the innate immune response [23]. Since their antimicrobial activity is mainly due to a membranolytic effect, the development of AMP-promoted microbial resistance appears less probable [24]. Insects possess many AMP families, with Cecropins (Cecs) representing the best-characterized group [25].

Recently, we characterized Q53 CecB, a natural CecB variant derived from the silkworm *Bombyx mori* [26,27]. In *B. mori*, Q53 CecB is synthetized as a 63-aa inactive form and matured via enzymatic cleavage in a 35-aa active peptide), characterized by a net charge of +7 [27]. In vitro studies demonstrated that Q53 CecB has an effective antimicrobial activity against the planktonic form of both mucoid and non-mucoid *P. aeruginosa* reference strains and low/absent toxicity against human cells in vitro [27]. Additionally, Q53 CecB maintains an anti-*Pseudomonas* activity at a high saline concentration, a typical condition of the mucus from patients affected by CF [28,29]. But, Q53 CecB is sensitive to protease degradation, as the peptide lost its antibacterial activity after a 2 h in vitro treatment with trypsin [27]. Susceptibility to proteolytic enzymes produced by both the host and pathogens represents one of the main limitations to a possible medical application of natural AMPs [30]. Nevertheless, this drawback can be limited in several ways, including the substitution of the natural L-amino acids with their respective D-enantiomers [31,32,33].

Here, we show that D-Q53 CecB, a synthetic Q53 CecB-derived isomer carrying D-amino acids throughout its entire sequence, had a stronger bactericidal activity against planktonic forms of mucoid and non-mucoid reference *P. aeruginosa* strains and it was highly resistant to in vitro enzymatic digestion with different proteases, compared to the natural L-peptide (hence L-Q53 CecB). Additionally, we display that D-Q53 CecB maintained its anti-*Pseudomonas* activity at high salt concentrations and in the presence of increasing concentrations of fetal-bovine serum, although at reduced levels. When tested in vitro against different types of human cells, D-Q53 CecB showed a general increment in toxicity levels compared to the L-isomer, but its bactericidal activity was at concentrations well below the range of cytotoxicity. Both L- and D-Q53 CecB peptides were active in inhibiting biofilm formation, but the D-enantiomer was extremely effective in inducing biofilm degradation, suggesting its possible use in an anti-*Pseudomonas* therapy.

## 2. Results

### 2.1. L- and D-Q53 CecB Sensitivity to Degradation by Human and P. aeruginosa Elastases

**Figure 1:**
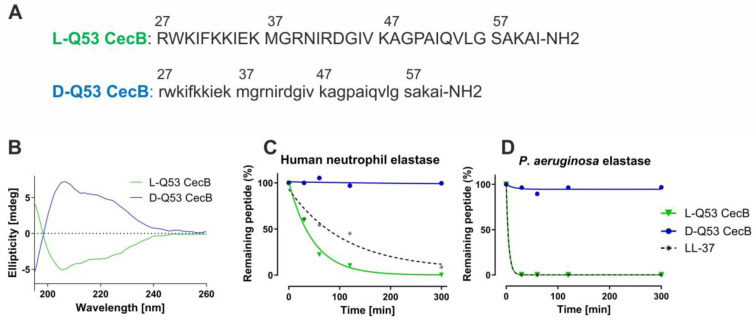
Effect of human neutrophil and *P. aeruginosa* elastases on L- and D-enantiomers of Q53 CecB. (**A**) L-Q53 CecB (green) and D-Q53 CecB (blue) mature peptide sequences (from 27 to 61 residues). Upper- and lowercase letters indicate L- and D-residues, respectively; NH2 indicates the amide group. (**B**) CD spectra of L-Q53 CecB (green) and D-Q53 CecB (blue) peptides at a concentration of 10 µM in 30% TFE. (**C**,**D**) Percentages (%) of intact peptide after incubation for 30, 60, 120, and 300 min with human neutrophil elastase (**C**) and *P. aeruginosa* elastase (**D**). Amounts of each peptide were determined using the integration analysis of the RP-HPLC profile. L-Q53 CecB (green continuous line); D-Q53 CecB (blue continuous line); LL-37 (reference peptide control, gray dotted line).

The L- and D-Q53 CecB peptides, both amidated in their C-terminus [34] (Figure 1A) were checked for their conformations in 30% trifluoroethanol (TFE) via circular dichroism (CD) analyses. CD spectra of the two peptides were mirror images, showing identical ellipticities but opposite signs (Figure 1B), confirming that D-Q53 CecB was the D-enantiomer of the L-peptide. Both peptides were subsequently analyzed for their in vitro sensitivity to proteolytic digestion by human and *P. aeruginosa* elastases, chosen as representative enzymes among the active proteases produced by the host and pathogen in the lung of patients affected by *P. aeruginosa* pulmonary infections [30]. The human cathelicidin-related peptide LL-37 was processed in parallel as a reference control. Each peptide was incubated with commercially available human or *P. aeruginosa* elastases for a total period of 5 h, and enzymatic degradation was analyzed by RP-HPLC at specific intervals of time (30, 60, 120, 300 min) (Figure 1C,D, Figures S1 and S2). Both enzymes were able to induce an almost complete degradation of L-Q53 CecB and LL-37 control peptides within 5 h. After 5 h incubation with the human neutrophil elastase, 0.3 and 8.7% of intact L-Q53 CecB and LL-37 peptides were identified, respectively. *P. aeruginosa* elastase showed a more efficient degradation activity on the same L-Q53 CecB and LL-37 peptides, as after 30 min incubation only ~0.2% of full-length peptide was observed in both cases. On the contrary, D-Q53 CecB was highly resistant to degradation, as ~ 96–99% of intact peptide was detected after 5 h treatment with both human and *P. aeruginosa* elastases (Figure 1C,D, Figures S1 and S2).

### 2.2. L- and D-Q53 CecB Antimicrobial Activity against P. aeruginosa Planktonic Bacteria

Previously we showed that L-Q53 CecB has a membranolytic activity against the planktonic forms of two reference strains of *P*. *aeruginosa* (non-mucoid: ATCC 27853; mucoid: ATCC 25668), with minimum inhibitory concentration (MIC) and minimal bactericidal concentration (MBC) values of 2.2 and 11 μM, respectively [27]. We, therefore, determined the anti-*Pseudomonas* activity of D-Q53 CecB in comparison to the L- counterpart *versus* the planktonic forms of the same ATCC 27853 and ATCC 25668 strains, and a third POA1-L strain (Lausanne subline) [35]. In all strains, the two peptides showed the same MIC values (2.2 μM; Table 1). However, the D-enantiomer appeared to be more effective against the three strains than the L-isomer when the MBC was examined. In particular, D-Q53 CecB showed MBC values of 4.4 μM with *P*. *aeruginosa* ATCC 27853 and ATCC 25668 strains and 2.2 μM in the presence of POA1L strain (Table 1). However, the POA1L displayed a higher sensitivity also when treated with the L- isomer, with an MBC of 4.4 μM (Table 1). As an antibiotic control, we used tobramycin, which showed the same MIC values in all the strains (2.14 μM; Table 1), but variable MBCs, resulting in less activity against POA1L than the other two strains (Table 1). A strain-dependent variability in AMP or conventional antibiotic sensitivity has already been reported for reference strains as well as clinical isolates of *P*. *aeruginosa* [36,37,38] and is due to the high phenotypic plasticity characteristic of this bacterium [39].

### 2.3. Effects of Cations, Serum, and Heat Treatments on the L- and D-Q53 CecB Anti-Pseudomonas Activity

It has been demonstrated that the anti-*Pseudomonas* activity of conventional antibiotics like tobramycin can be antagonized by several components present in the lung environment or in biological fluids [40,41]. Since similar conditions can also modify the AMP effectiveness [42,43,44], we evaluated the in vitro antibacterial activity of L- and D-Q53 CecB peptides at increasing concentrations of different cations and fetal bovine serum (FBS), using *P. aeruginosa* ATCC 27853 as a reference strain (Table 2). L- and D-Q53 CecBs retained their anti-*Pseudomonas* activity, albeit the MIC values of one or both peptides increased in some conditions (Table 2). In 100 mM NaCl, L- and D-Q53 CecB peptides maintained their MICs at 2.2 μM; while in 200 mM NaCl, D-Q53 CecB resulted less effective compared to the L-counterpart (4.4 μM; Table 2). At and over 300 mM NaCl, the two peptides were similarly less active (Table 2). In the presence of divalent ions Ca^2+^ and Mg ^2+^, the peptide efficiency was mostly reduced at Ca^2+^ concentrations of 2 mM, with both L- and D-Q53 CecB peptides showing MIC values of 15 μM (Table 2). Differently from the L-isomer, D-Q53 CecB decreased its activity also in the presence of Mg^2+^, as its MIC was 4.4 μM at 1 mM MgCl_2_. However, when tested at 0.5 mM MgCl_2_, both peptides showed the same MIC value of 2.2 μM as without Mg^2+^ (Table 2). Both enantiomers were active at 10 and 20% FBS although at higher concentrations than those observed in the absence of FBS (Table 2). Finally, we showed that similarly to the L-native peptide, the D-enantiomer was temperature stable, as pretreatments at 100 °C from 5 to 30 min did not affect its antimicrobial activity (Table 2).

### 2.4. L- and D-Q53 CecB Inhibitory Effects on P. aeruginosa Biofilm Formation

**Figure 2:**
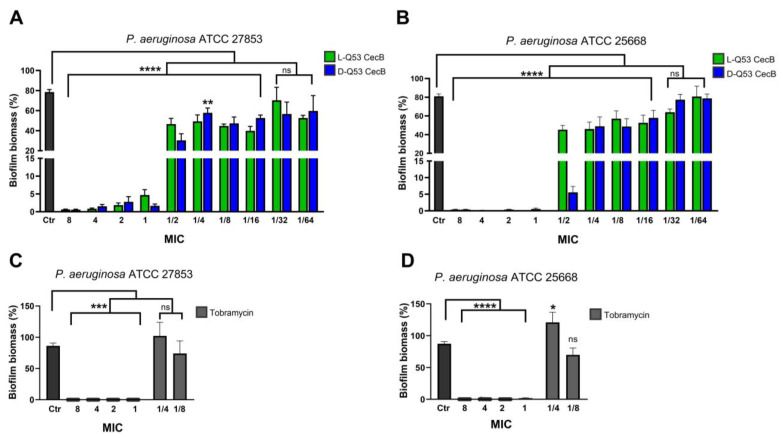
L- and D-Q53 CecB peptides inhibit *P. aeruginosa* biofilm formation. Inhibition of *P. aeruginosa* biofilm formation after a 24 h incubation with increasing concentrations of (**A**,**B**) L-Q53 CecB (green), D-Q53 CecB (blue), and (**C**,**D**) tobramycin (light gray), expressed as MIC folds [in (**A**,**B**): 1 MIC = 2.2 µM; in (**C**,**D**): 1 MIC = 2.14 µM]. Biofilm biomass (mean % ± SEM) in (**A**,**C**) ATCC 27853, non-mucoid strain, and (**B**,**D**) ATCC 25668, mucoid strain. One-way ANOVA showed that L- and D-enantiomers, as well as tobramycin, significantly inhibited biofilm formation in both strains ((**A**): F_20,191_ = 45.25, *p* < 0.0001; (**B**): F_20,207_ = 50.14, *p* < 0.0001; (**C**): F_6,59_ = 13.55, *p* < 0.0001; (**D**): F_6,64_ = 34.69, *p* < 0.0001). In (**A**–**D**), post-hoc Dunnet’s tests revealed significant reductions in treated samples compared to untreated controls (Ctr; dark gray). ****: *p* < 0.0001; ***: *p* < 0.001; **: *p* <0.01; *: *p* < 0.05; ns: non-significant differences for comparisons between treated samples and untreated controls (*p* > 0.05).

L- and D-Q53 CecB peptides were assessed for their ability to inhibit *P*. *aeruginosa* biofilm formation using the least peptide-sensitive non-mucoid (ATCC 27853) and mucoid (ATCC 25668) strains. For both strains, bacteria were statically incubated for 24 h with increasing concentrations of L- or D-Q53 CecBs, from 1/64 (0.034 μM) to 8 times the MIC (17.6 μM). As a control, inhibition of biofilm formation was evaluated in the presence of increasing concentrations of tobramycin, from 1 MIC (2.14 μM) to 8 MIC (17.12 μM). Additionally, the effect of tobramycin at two sub-MIC concentrations (1/8 and 1/4 MIC) was assessed. Biofilm biomass levels were quantified in comparison to those of untreated controls using crystal violet assays (Figure 2).

Analysis of variance indicated that both enantiomers were able to inhibit *P. aeruginosa* biofilm formation (*p* < 0.0001 in both ATCC 27853 and ATCC 25668 strains). L- and D-Q53 CecB peptides showed similar and strong inhibitory activities for concentrations from 8 to 1 MIC, with significant 99.9–94% decrements in biofilm biomass, in comparison to untreated controls (Figure 2A,B). Moreover, both L- and D- isomers were able to significantly reduce biofilm formation when tested at sub-MIC concentrations from 1/2 to 1/16 MIC, with decrements in biofilm biomass ranging from ~30 to 60% in the non-mucoid strain and from ~30 to 44% in the mucoid strain (Figure 2A,B). The only exception was represented by 1/2 MIC D-Q53 CecB-treated samples which displayed a 93% mean decrement with respect to untreated controls in the mucoid strain. At 1/32 and 1/64 MIC, L- and D-peptides did not show any significant inhibitory effect on biofilm formation in both *P*. *aeruginosa* strains in comparison to untreated controls (Figure 2A,B).

Tobramycin was also able to significantly inhibit *P. aeruginosa* biofilm formation (*p* < 0.0001 in both ATCC 27853 and ATCC 25668; Figure 2C,D). In both strains, this compound was effective in inhibiting ~99.9% of biofilm at concentrations from 8 to 1 MIC, while differently from Cec B enantiomers, it was not active at sub-MIC concentrations. In particular, in samples treated with 1/4 MIC tobramycin, biofilm biomass was not affected in ATCC 27853 strain and was significantly higher in comparison to untreated controls in ATCC 25668 strain. In both *P. aeruginosa* strains, samples treated with 1/8 MIC tobramycin showed a % of biofilm biomass not significantly different from that of untreated controls (Figure 2C,D).

### 2.5. L- and D-Q53 CecB Degradation Activities on Pre-Formed P. aeruginosa Biofilm

**Figure 3:**
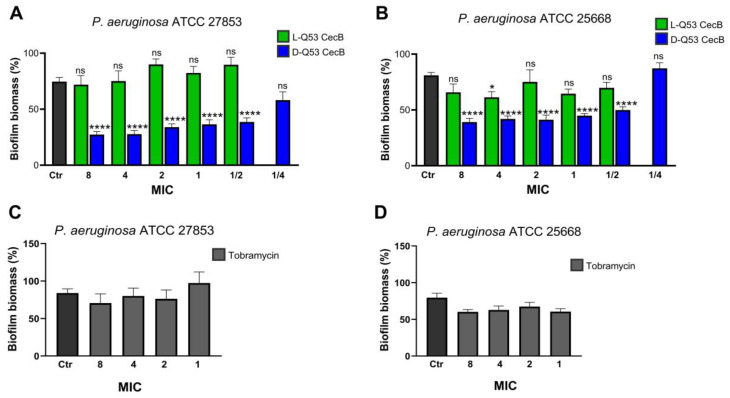
Effects of L- and D-Q53 CecB treatments on *P. aeruginosa* pre-formed biofilm. Biofilm biomass (mean % ± SEM) of 24 h old *P. aeruginosa* biofilms after 1 h treatment with increasing concentrations of (**A**,**B**) L-Q53 CecB (green), D-Q53 CecB (blue), and (**C**,**D**) tobramycin (light gray), expressed as MIC folds [in (**A**,**B**): 1 MIC = 2.2 µM; in (**C**,**D**): 1 MIC = 2.14 µM]. % Biofilm biomass in (**A**,**C**) ATCC 27853, non-mucoid strain, and (**B**,**D**) ATCC 25668, mucoid strain. In (**A**,**B**): one-way ANOVA showed significant reductions in biofilm biomass in both strains (ATCC 27853: F_11,79_ = 21.51, *p* < 0.0001; ATCC 25668: F_11,192_ = 11.38, *p* < 0.0001). In (**A**,**B**): post-hoc Dunnett’s test detected significant biofilm biomass decrements in samples treated with D-Q53 CecB from 8 to 1/2 MIC and in a single sample treated with 4 MIC L-Q53 CecB in ATCC 25668, in comparison to untreated controls (Ctr; dark gray). All the other comparisons between L- or D-Q53 CecB-treated samples and negative controls (Ctr; dark gray) did not show any significant difference in biofilm biomass reduction. ****: *p* < 0.0001; *: *p* < 0.05; ns: non-significant (*p* > 0.05). In (**C**,**D**): one-way ANOVA showed that 1 h tobramycin treatments did not induce any significant variation in biofilm biomass in both strains (ATCC 27853: F_4,32_ = 1.05, *p* = 0.57; ATCC 25668: F_4,35_ = 2.53, *p* = 0.6).

L- and D-Q53 CecB peptides were tested for their ability to degrade mature biofilms using the same *P*. *aeruginosa* non-mucoid and mucoid strains. Twenty-four hour-old biofilms were incubated for 1 h with increasing concentrations of L- and D-Q53 CecB peptides, from 1/2 MIC (1.1 μM) to 8 MIC (17.6 μM). D-Q53 CecB was additionally tested at 1/4 MIC (0.55 μM). Pre-formed biofilms were also treated for 1 h with increasing concentration of tobramycin, from 1 MIC (2.14 μM) to 8 MIC (17 μM). Untreated controls were grown in parallel, and biofilm biomasses were quantified in crystal violet assays (Figure 3).

We detected a significant reduction in % of biofilm biomass after treatment with D- Q53 CecB (Figure 3A,B; *p* < 0.0001 in both ATCC 27853 and ATCC 25668 strains). The mean decrements in the different D-Q53 CecB-treated samples ranged from 64 (8 MIC) to 49% (1/2 MIC) in the non-mucoid strain and from 52 (8 MIC) to 38% (1/2 MIC) in the mucoid strain. D-Q53 CecB lost its ability to reduce pre-formed biofilms at concentrations of 1/4 MIC (0.55 μM) (Figure 3A,B). On the contrary, the native L-Q53 CecB peptide at concentrations from 1/2 to 8 MIC did not significantly reduce biofilm biomass compared to untreated controls, except for a single sample in the mucoid strain at 4 MIC (Figure 3A,B). In both strains, 1 h treatment with tobramycin was not efficient in reducing 24 h old biofilm at all tested concentrations, from 1 to 8 MIC (Figure 3C,D).

To determine whether the biofilm reduction in D-Q53 CecB samples was due to a peptide bactericidal activity or a detachment of living bacterial cells from pre-formed biofilms, we assessed the number of live bacteria in the medium after 1 h treatment with D-Q53 CecB at 1 MIC (2.2 μM), performing a Colony Forming Units (CFUs) assay. The L-peptide was evaluated in the same conditions for comparison. Culture media deriving from both L- and D-Q53 CecB samples showed strong reductions in viable cells with respect to untreated controls. D-Q53 CecB treatments induced mean CFUs/mL decrements of 4 and 7 orders of magnitude in the non-mucoid (ATCC 27853) and mucoid (ATCC 25668) strains, respectively. In L-Q53 Cec B samples, mean CFUs/mL decrements were 6 and 10 orders of magnitude in the non-mucoid and mucoid strains, respectively (Table 3).

### 2.6. Exploring L- and D-Q53 CecB Mechanisms Inhibiting P. aeruginosa Biofilm Formation

#### 2.6.1. In Vitro Interaction with DNA

**Figure 4:**
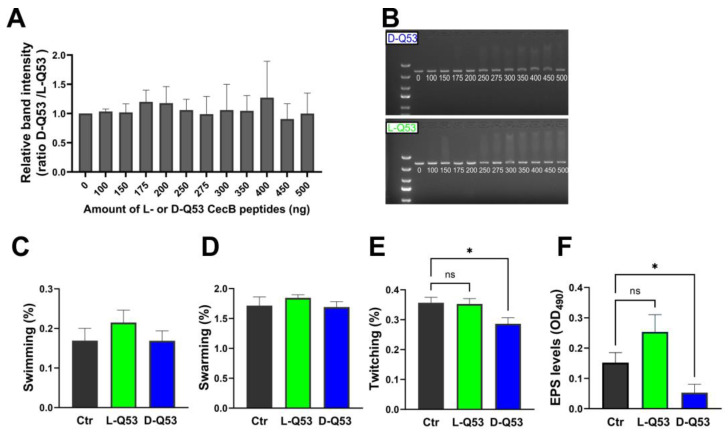
Effects of L- and D-Q53 CecB peptides on in vitro DNA mobility, bacterial motility, and EPS production. (**A**,**B**) In vitro interactions between DNA and increasing amounts of CecB peptides**.** (**A**) Ratio (mean ± SEM) between the DNA band intensities reduction obtained with the same amount of D-and L-Q53 CecB peptides. One-way ANOVA revealed non-significant differences in the interaction properties of the two peptides (F_11,32_ = 0.11, *p* = 0.99). (**B**) Representative images of gel retardation assays showing increasing band smearing when linearized DNA was incubated with increasing amounts of D-Q53 CecB (D-Q53, blue; upper part) and L-Q53 CecB (L-Q53, green; lower part). Numbers in gel lanes indicate the amount of peptide (ng). (**C**–**F**) Bacterial motilities and EPS levels in *P. aeruginosa* ATCC 25668 strain, in the presence of sub-MIC concentrations of L- and D-peptides, not affecting bacterial growth (see text for details). (**C**) Swimming: % of the growing area/plate area (mean ± SEM) in 0.3% agar plates. One-way ANOVA revealed non-significant differences among samples (F_2,6_ = 0.81, *p* = 0.49). (**D**) Swarming: % of the growing area/plate area (mean ± SEM) in 0.5% agar plates. One-way ANOVA revealed non-significant differences among samples (F_2,6_ = 0.65, *p* = 0.55). (**E**) Twitching: % of the growing area/plate area (mean ± SEM) in 1% agar plates. *, *p* < 0.05: significantly reduced twitching in D-Q53 CecB samples compared to untreated controls (F_2,9_ = 4.37, *p* < 0.05; post hoc Dunnett’s test: *p* < 0.05). (**F**) EPS levels (mean OD_490_ ± SEM). *, *p* < 0.05: significantly reduced EPS levels in D-Q53 CecB samples compared to untreated controls (F_2,12_ = 18.16, *p* < 0.05; post hoc Dunnett’s test: *p* < 0.05). Ctr (gray): Controls; L-Q53 (green): L-Q53 CecB-treated samples; D-Q53 (blue): D-Q53 CecB-treated samples. ns: non-significant (*p* > 0.05).

The possible interaction of L- and D-Q53 CecB peptides and eDNA was evaluated by using linearized DNA as a proxy. We previously showed that the L-Q53 CecB isomer can interact with DNA in vitro [27]. To determine whether the D-Q53 CecB isomer displayed similar characteristics, we performed a gel retardation assay comparing the DNA binding activities of L- and D-peptides processed in parallel. Purified DNA was incubated with increasing amounts of L- or D-peptides and loaded into a 1% agarose gel. The two peptides showed similar binding properties to DNA, as the ratio between the DNA band intensities for D-and L-Q53 CecBs resulted in ~1 in all tested conditions (Figure 4A,B).

#### 2.6.2. Peptides’ Effects on Bacterial Motility and EPS Production

The reduction in biofilm formation by L- and D-Q53 CecB peptides at sub-MIC concentrations ranging from 1/2 to 1/16 MIC suggested that the peptides’ inhibitory effect was not simply due to a decrease of bacterial growth but to a possible ability of these AMPs to interfere with the different processes acting during the early phases of biofilm formation, including bacterial motility and EPS production. Therefore, we first evaluated the growth curves of planktonic *P. aeruginosa* in the presence of variable concentrations of either L- or D-Q53 CecB ranging from 1 MIC (2.2 μM) to 1/8 MIC (0.275 μM). We established that 1/2 MIC for L-Q53 CecB and 1/4 MIC for D-Q53 CecB were the sub-MIC concentrations not inhibiting bacterial growth in both *P. aeruginosa* strains (Figure S3). Since the two *P. aeruginosa* strains showed similar behaviors in the presence of both CecB peptides, we focused the analyses on the mucoid strain (ATCC 25668).

The ability of L- and D-Q53 CecB peptides to impact swimming, swarming and/or twitching motilities was examined by evaluating the bacterial growth in Luria Bertani (LB) plates, supplemented with 1/2 MIC L-Q53 CecB or 1/4 MIC D-Q53 CecB, and respectively containing 0.3, 0.5, and 1% agar, as in [36,45] (Figure 4C–E and Figure S4). In 0.3 and 0.5% agar conditions, both enantiomers did not significantly alter the bacterial growth area compared to the relative controls, indicating they were not able to affect swimming and swarming motilities (Figure 4C,D). However, D-Q53 CecB was able to impact twitching, as significant reductions of bacterial growth areas were detected in D-peptide-supplemented 1% agar plates compared to negative controls (Figure 4E; *p* < 0.05). On the contrary, the L-peptide had no effect on twitching (Figure 4E).

We evaluated the effects of L- and D-Q53 CecBs on *P. aeruginosa* EPS production, measuring water-soluble EPS levels in planktonic bacteria, following the procedure by [46]. After a 20 h incubation with L- and D-Q53 CecBs, at their respective sub-MIC concentrations in 1% sucrose-supplemented LB medium, EPSs were spectrophotometrically quantified and compared to those of negative controls, using the phenol-sulfuric acid method. D-Q53 CecB was able to significantly reduce EPS production in comparison to untreated controls (Figure 4F; *p* < 0.05). On the contrary, the L-peptide did not show any significant effect (Figure 4F).

#### 2.6.3. L- and D-Q53 CecB Effects on Transcription of Representative Genes Involved in Biofilm Formation

**Figure 5:**
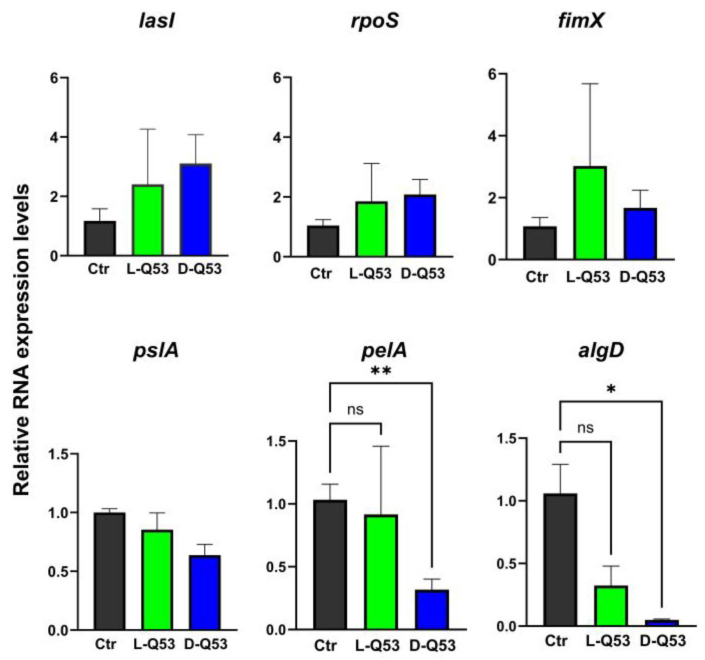
Effects of L- and D-peptides on transcription of *P. aeruginosa* representative genes involved in biofilm formation, twitching motility, and EPS production. Relative transcription levels (mean fold change ± SEM) in *P. aeruginosa* (ATCC 25668) samples treated with L-Q53 CecB (L-Q53; green), D-Q53 CecB (D-Q53; blue), and in negative controls (Ctr; gray) of the following genes: *lasI*, *rpoS*, *fimX*, *pslA*, *pelA*, *algD.* Kruskal–Wallis tests revealed not significant differences among samples in the expression levels of *lasI*, *rpoS*, *fimX*, and *pslA* (*p* > 0.05). *pelA* and *algD* resulted significantly downregulated in D-Q53 CecB-treated samples compared to negative controls (*pelA:* Kruskal–Wallis test *p* < 0.01; post-hoc Dunn’s test: *p* < 0.01 (**); *algD*: Kruskal–Wallis test *p* < 0.05; post-hoc Dunn’s test: *p* < 0.05 (*); ns, non-significant).

We assessed whether CecB peptides at sub-MIC concentrations were able to affect the expression of some representative *P. aeruginosa* genes known to be involved in the initial phases of biofilm development, twitching motility, and EPS production. A 20 h incubation with 1/2 MIC L- and 1/4 MIC D-Q53 CecB peptides did not significantly alter expression levels of *lasI*, coding a quorum sensing molecule (acyl-homoserine–lactone synthase), known to enhance the expression of several bacterial genes involved in biofilm formation [47], and *rpoS*, specifying an alternative sigma factor (RpoS) regulating the bacterial stress response and involved in different aspects of *P. aeruginosa* biofilm biogenesis, including *Psl* transcription [48,49] (Figure 5). Similarly, the expression levels of *fimX,* encoding a multidomain protein (FimX) important in the regulation of Type IV pilus assembly and twitching motility [50,51,52], were not significantly different in L- and D-Q53 CecB-treated samples compared to negative controls (Figure 5). Additionally, we determined the transcriptional levels of *pslA*, *pelA*, and *algD*, as representative genes of the *Psl*, *Pel*, and *alginate* operons, respectively, which are actively transcribed during *P. aeruginosa* EPS production [13,53]. Twenty h treatments with L- and D-Q53 CecB peptides did not affect *pslA* transcription (Figure 5), whereas we found a significantly lower expression of *pelA* and *algD* in D-Q53 CecB-treated samples than in negative controls (Figure 5).

### 2.7. Exploring L- and D-Q53 CecB Effects on Pre-Formed P. aeruginosa Biofilm

**Figure 6:**
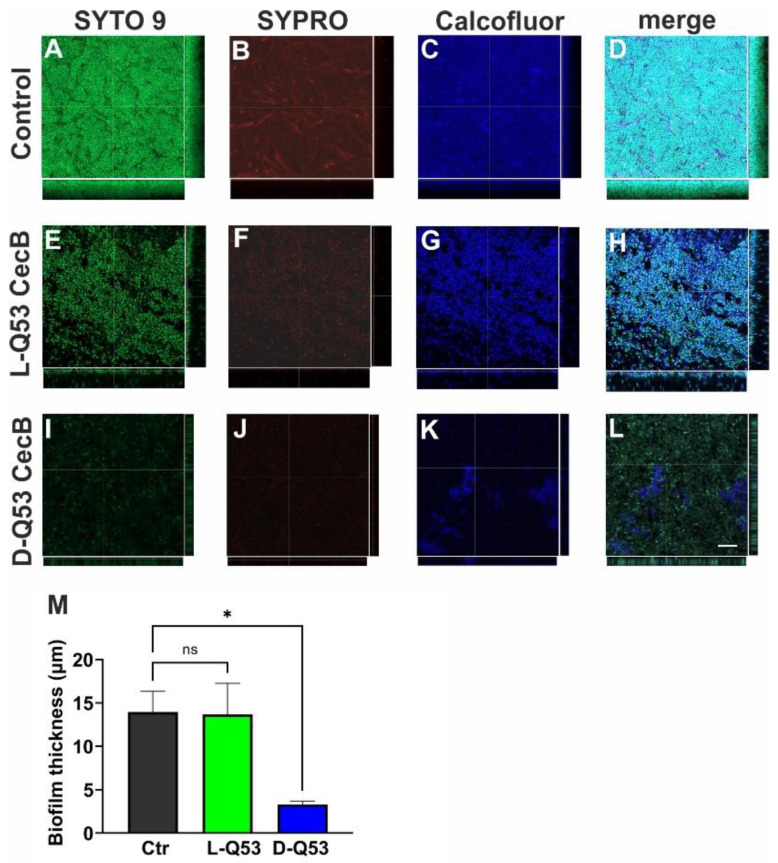
*P. aeruginosa* biofilm structure after 1 h treatment with L-Q53 CecB or D-Q53 CecB peptides. Moreover, 24 h old *P. aeruginosa* biofilms (ATCC 25668) treated with 2.2 µM L-Q53 or D-Q53 CecB peptides for 1 h and stained with SYTO™ 9 (staining bacteria; green), SYPRO™ Ruby Biofilm Matrix Stain (protein staining; red) and Calcofluor White (EPS biofilm matrix staining; blue). Representative images (top view (x-y plane) and cross-section views (bottom: x-z plane; right: y-z plane) of (**A**–**D**) untreated biofilm control; (**E**–**H**) L-Q53 CecB-treated biofilm; (**I**–**L**) D-Q53 CecB-treated biofilm. Bar in L corresponds to 10 μm. (**M**) Biofilm thickness (mean ± SEM) in untreated control (Ctr; gray) and after 1 h treatment with 2.2 µM L-Q53 CecB (green) or 2.2 µM D-Q53 CecB (blue), evaluated in 6–8 independent confocal fields per condition. Analysis of variance revealed a significant thickness reduction in D-Q53 CecB-treated biofilms compared to untreated controls (F_2,17_ = 5.6, *p* < 0.05; post-hoc Dunnett’s test *p* < 0.05). *: *p* < 0.05; ns: non-significant.

To better understand the effect of CecB peptides on mature biofilms, we performed a confocal microscope analysis. Since the two *P*. *aeruginosa* strains displayed similar responses in the crystal violet assay, we focused on the mucoid strain, known to possess a strong alginate-rich mature biofilm. After a 1 h treatment with 1 MIC (2.2 μM) of L- or D-Q53 CecBs, 24 h old biofilms were stained with SYTO^TM^ 9 (green), SYPRO^TM^ (red), and Calcofluor White (blue), to respectively visualize embedded bacterial cells, proteins, and EPSs of the biofilm. Negative controls were processed in parallel (Figure 6). Additionally, the effect of treatments on biofilm thickness was estimated by measuring the biofilm width on the *Z*-axis in six/eight independent confocal fields per condition (Figure 6M). As expected, untreated biofilms showed a thick and defined structure, with bacterial cells encased within a well-organized ECM (Figure 6A–D,M). At the ECM level, both proteins and EPS components showed a gradient of distribution, with a stronger signal in the basal region of the biofilm (in proximity to the slide surface) and a weaker staining in the distal part of the biofilm (Figure 6A–D,M). A 1 h treatment with L-Q53 CecB peptide did not significantly reduce biofilm thickness in comparison to untreated controls (Figure 6M). However, the L-peptide seemed able to slightly alter the biofilm structure since in L-Q53 CecB samples, bacteria appeared less densely packed and the ECM less stained in both protein and EPS components compared to untreated controls (Figure 6E–H). On the other hand, D-Q53 CecB was able to strongly affect biofilm structure and organization. In fact, D-Q53 CecB-treated samples showed a ~77% significant reduction in biofilm thickness with respect to untreated controls (Figure 6M; *p* < 0.05). Moreover, in the remaining biofilm, ECM architecture appeared strongly compromised, with both proteins and EPSs weakly stained (Figure 6I–L).

**Figure 7:**
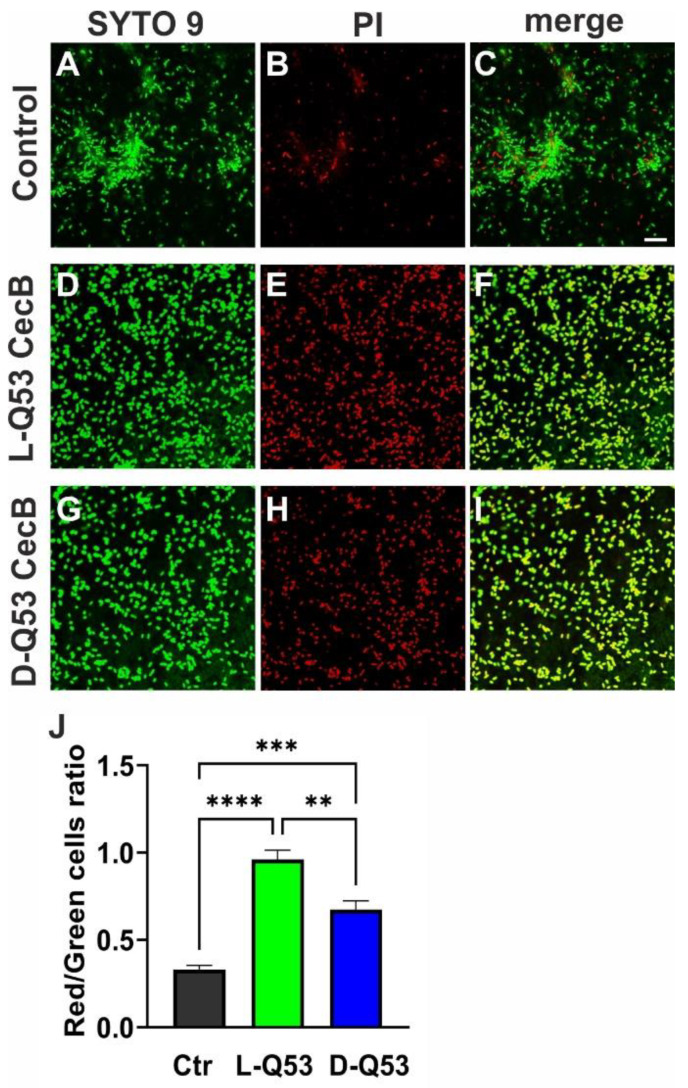
*P. aeruginosa* biofilm-embedded cells after 1 h treatment with L-Q53 CecB or D-Q53 CecB peptides. 24 h old *P. aeruginosa* biofilms (ATCC 25668) treated with 2.2 µM L-Q53 or D-Q53 CecB peptides for 1 h and strained with SYTO™ 9 (staining bacteria; green) and Propidium Iodide (dead bacteria; red). Images are ~0.5 μm Z-projections of 4 confocal sections. Representative images of (**A**–**C**) untreated biofilm control; (**D**–**F**) L-Q53 CecB-treated biofilm; (**G**–**I**) D-Q53 CecB-treated biofilm. Bar corresponds to 5 μm. (**J**) Ratio of red/green cells (mean ± SEM) in untreated control (Ctr; gray) and after 1 h treatment with 2.2 µM L-Q53 CecB (green) or D-Q53 CecB (blue), evaluated in at least 24 independent confocal sections per condition. Kruskal-Wallis test followed by post-hoc Dunn’s test revealed a significant increment in red/green ratio in both L- and D-Q53 CecB-treated biofilms compared to untreated biofilms, as well as in L- compared to D-Q53 CecB-treated samples (****: *p* < 0.0001; ***: *p* < 0.001; **: *p* < 0.01; N of counted cells: untreated control: 5081; L-Q53 CecB: 2787; D-Q53 CecB: 3986).

We finally determined whether CecB peptides were able to affect the vitality of the remaining biofilm-embedded bacteria. Via a confocal microscope analysis, we estimated life and dead bacteria after 1 h treatment with 1 MIC of L- or D-Q53 CecBs on 24 h-old biofilms stained with SYTO 9 and Propidium Iodide, to respectively detect total (green) and dead (red) cells. Untreated biofilms contained a certain number of dead cells, which appeared to increase in both L- and D-Q53 CecB-treated samples (Figure 7A–I). To quantitatively evaluate the killing effects of the peptides, we determined the ratio between red and green cells at the level of the upper parts of the biofilm, as these regions had certainly been in contact with the peptide in CecB-treated samples. Significant increments in red/green cell ratio were observed in both L- and D-Q53 CecB samples in comparison to untreated controls. Moreover, the mean red/green cells ratio in samples treated with L-Q53 CecB was significantly higher than those treated with the D-enantiomer (Figure 7J), suggesting that both peptides exert a killing activity on biofilm-embedded cells, with higher efficiency of the L-isomer compared to the D-counterpart.

### 2.8. L- and D-Q53 CecB Hemolytic and Cytotoxic Activity

**Figure 8:**
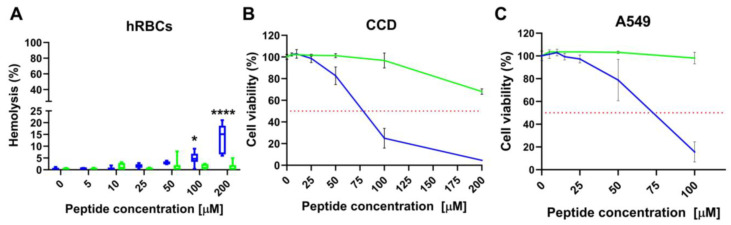
Hemolytic and cytotoxic activities of L- and D-Q53 CecB peptides. (**A**) Hemolysis (median ± 10–90 percentiles) of human red blood cells (hRBCs) after incubation with increasing concentrations of L-Q53 CecB (green) and D-Q53 CecB (blue). Significant hemolysis was detected only in samples treated with D-Q53 CecB at 100 and 200 μM (Mixed-effects model: concentration X peptide interaction, *p* < 0.0001; * and ****: *p* < 0.05 and *p* < 0.0001 in 100 and 200 μM D-Q53 CecB-treated samples, respectively, in a post hoc Sidak multiple comparisons test). (**B**,**C**) Cell viability (mean ± SD) of human lung fibroblasts (CCD-34Lu) (**B**), and human type II pulmonary epithelial cells (A549) (**C**), after incubation with increasing concentrations of L-Q53 CecB (green) and D-Q53 CecB (blue).

To assess the toxicity potential, we analyzed the L- and D-Q53 CecB peptides for their hemolytic activity in human red blood cells (hRBCs), as well as their cytotoxicity against a human lung fibroblast cell line (CCD-34Lu) and a reference model for Type II alveolar epithelial cells (A549) [54] (Figure 8). L-Q53 CecB did not display any significant hemolytic effect for concentrations up to 200 μM. D-Q53 CecB showed negligible hemolytic activity (i.e., less than 5%) up to 50 μM and a minimum hemolytic concentration (MHC) at 100 μM, with mean hemolysis levels of 5.9%. At 200 μM, the D-peptide displayed a further slight increment in hemolytic activity (13.6%; Figure 8A and Table 4). Cytotoxicity experiments on CCD-34Lu and A549 cells, incubated for 24 h with increasing concentrations of L- and D-Q53 CecB peptides, showed that the natural L-isomer did not possess any evident toxic effect for concentrations up to 100 μM in both cell lines, while a ~30% cell viability reduction was detected at 200 μM in CCD-34Lu primary fibroblasts (Figure 8B,C and Table 4). Compared to the L-isomer, D-Q53 CecB showed higher cytotoxicity in both cell lines, with half maximal inhibitory concentrations (IC_50_) at ~73 and 68 μM in CCD-34Lu and A549 cell lines, respectively (Figure 8B,C and Table 4). In both cases, the D-peptide did not display any cytotoxic effect for concentrations up to 25 μM while determining a progressive decrease in cell viability at higher concentrations (CCD-34Lu fibroblasts: ~85, 27 and 4.5% at 50, 100, and 200 μM, respectively; A549 cells: ~79 and 15% at 50 and 100 μM, respectively) (Figure 8B,C).

The selectivity index (SI), calculated as the ratio between the mean IC_50_ and MBC values, was used to assess the peptides’ cell selectivity between bacteria and host cells. Higher SIs indicate a greater cell selectivity; however, as SIs ≥ 10 and ≥3 have both been reported as promising values for effective drugs, a minimum acceptable SI has not yet been clearly established [55]. In CCD-34Lu fibroblasts, we obtained SI values against *P. aeruginosa* > 18 and 16.47 for L- and D-Q53 CecB, respectively. In A549 cells, SIs were >9 for the L-form and 15.45 for the D-enantiomer (Table 4).

## 3. Discussion

We previously showed that the natural *B. mori* L-Q53 CecB isomer has a remarkable anti-*Pseudomonas* activity and a low/absent toxicity against human cells, suggesting this peptide as a good candidate for the development of compounds to treat *Pseudomonas* lung infections [27]. Despite these promising characteristics, L-Q53 CecB was sensitive to enzymatic degradation. We, therefore, produced the D-Q53 CecB enantiomer, which was completely resistant to 5 h enzymatic digestion by both human neutrophil and *P. aeruginosa* elastases, confirming that a D-enatiomerization protects the AMP from proteolytic digestion [33,43,56,57].

Pioneering studies have shown that D-enantiomeric forms of AMPs known to disrupt the bacterial membrane, such as Cecs, maintain their antimicrobial activity [33,56,57]. When we compared the L- and D-Q53 CecB anti-*Pseudomonas* activity, we showed that the two peptides possessed a similar bacteriostatic activity, but D-Q53 CecB had a higher bactericidal efficiency, with ~50–60% lower MBC values than the L- variant against *P. aeruginosa* planktonic bacteria. These results were likely due to the resistance of the D-enantiomeric form to digestion by *Pseudomonas* proteases.

D-Q53 CecB retained its antibacterial activity at NaCl concentrations that can be found in the lung environment of patients affected by CF (~130 mM), who frequently suffer from *P. aeruginosa* respiratory infections [29]. Although at reduced levels, D-Q53 CecB maintained its antibacterial activity in different environmental conditions, such as a high % of serum, used to mimic complex biological fluids [58], and elevated levels of Ca^2+^ and Mg^2+^, present in human saliva at 1–2 and 0.5 mM concentrations, respectively [42,59]. Increments in effective antibacterial concentrations in less favorable experimental conditions have already been reported for other AMPs [42,43,44,58]. From a perspective of a possible biomedical application of D-Q53 CecB, we performed a series of in vitro analyses to determine the peptide hemolytic activity in hRBCs and cytotoxicity against two different types of human lung cell lines. We detected an overall higher in vitro toxicity of the D-Q53 CecB isomer compared to the natural L-counterpart**,** likely due to the L- to D-enantiomer substitution, which was found to affect cytotoxicity against eukaryotic cells of other AMP derivates [60]. However, this higher toxicity should not hamper the development of D-Q53 CecB-based compounds for medical purposes. Indeed, the peptide’s MHC was respectively ~23- and 7-fold higher than its MBC and MIC in the most perturbing condition (2 mM CaCl_2_). Similarly, D-Q53 CecB IC_50_ values determined in both primary lung fibroblasts and a Type II pulmonary epithelial cell model were ~15- and 4-fold higher than the peptide’s MBC and MIC in 2 mM CaCl_2_, respectively. When we measured the D-Q53 CecB peptide selectivity between bacteria and host cells for both cell lines, we obtained SIs > 15, representing a promising value for effective drugs [55].

There is a general lack of compounds able to efficiently treat *P. aeruginosa* biofilms, often identified in patients affected by chronic infections [5,61,62]. In agreement with these findings, we showed that the conventional antibiotic tobramycin affected biofilm development when tested at concentrations equal to or higher than the MIC value but did not display any inhibitory effect at sub-MIC concentrations. Rather, this antibiotic promoted biofilm formation in specific sub-MIC conditions, as also previously described and deeply studied elsewhere [63,64]. On the contrary, L- and D-Q53 CecB peptides were able to prevent biofilm formation at concentrations identical to or greater than the MIC while reducing its development at sub-MIC concentrations from 1/2 to 1/16 MIC. A peptide capability to inhibit biofilm formation at sub-MIC concentrations has already been observed for other AMPs, like the Temporin-GHc and -GHd peptides against *Streptococcus mutans* [46] and the esculentin-derived Esc(1-21)-1c against *P. aeruginosa* [36]. In both cases, the inhibitory activity was associated with a peptide capacity to interfere with the complex bacterial processes occurring during biofilm development rather than a decrement in bacterial growth [36,46].

Different mechanisms can explain the inhibitory effects of L- and D-Q53 CecB peptides on biofilm formation.

In fact, the L- and D-isomers showed similar in vitro DNA binding activity, suggesting that the eDNA produced during biofilm formation might represent a possible target for both peptides through the interaction between negatively charged eDNA and positively charged L- or D-peptides. This mechanism of action may limit the promotion of cell-cell adhesion and aggregation to a surface, which are considered fundamental processes in the early phases of biofilm development [5,14].

Moreover, the D-Q53 CecB peptide appeared to have additional inhibitory effects on other processes important for biofilm development. The D-form was able to decrease the twitching motility and EPS production. This effect on twitching was not associated with any variation in the expression levels of *lasI*, coding a quorum-sensing factor modulating the expression of several genes involved in biofilm formation [47]. Similarly, the D-peptide did not affect RNA levels of *fimX*, a fundamental gene regulating Type IV pilus assembly and twitching motility [50,51]. As assembly and functional regulation of Type IV pili are complex processes involving multiple factors [50], further investigations will define the level at which D-Q53 CecB exerts its inhibitory role. We cannot exclude that the D-peptide impacts on transcription of other genes involved in the biogenesis of the pilus, and only an extensive transcriptomic study will clarify this aspect. D-Q53 CecB inhibitory effect on EPS production may occur at a transcriptional level as we observed a decrease in the expression of *pelA* and *algD*, which are respectively involved in the production of Pel and alginate, two out of the three main components of *P. aeruginosa* EPS. The reduction in *pelA* and *algD* expressions appeared specific, as neither *pslA*, belonging to the *Psl* operon, nor *rpoS*, coding an alternative sigma factor important in several bacterial processes, including *Psl* transcription [48,49], were affected by D-Q53 CecB treatments.

None of the analyzed genes were altered by treatments with the L-counterpart, which exerts its antibacterial effect through a membranolytic activity [27]. It is widely accepted that the D-forms of AMPs like Cecs, known to target and disrupt bacterial membranes, have an action mechanism similar to that of the native L-peptide because they are mirror images of each other and interact with achiral components at the membrane levels [31,33,56]. However, it has also been demonstrated that the L- and D-peptides can interact with some molecular targets differently, like with LPS, which carries both chiral and achiral portions in its molecular structure [57]. The differences in the ability of L- and D-Q53 CecB forms to interfere with the transcription may be due to a variable interaction with yet unidentified chiral molecular targets. Although further analyses are required, we might hypothesize that these putative D-Q53 CecB targets are localized at a membrane level, as our previous analyses did not show any evident localization of the L-enantiomer in the cytoplasm [27]. These interactions could induce a response mediated by second messengers involved in biofilm formation, like guanosine tetra- and pentaphosphate and/or bis-(3′-5′)-cyclic dimeric GMP [36,65,66]. Although we did not see any detectable difference between the two enantiomers in inhibiting biofilm biomass in the crystal violet assay, these mechanisms could explain the additional D-Q53 CecB activities, thus strengthening the D-peptide contrasting effects against *Pseudomonas* biofilms.

A further critical feature in fighting *P. aeruginosa* infections is the eradication of pre-formed biofilms. Our data confirmed that the common antibiotic tobramycin has a negligible biofilm degradation efficiency, a characteristic previously associated with the ability of distinct ECM components to reduce antibiotic penetration of *P. aeruginosa* biofilms [67,68]. In contrast, we showed that a 1 h treatment with D-Q53 CecB was able to degrade 24 h old biofilms, reducing biomass and affecting the whole organization, strongly compromising both protein and EPS components of the ECM. The biofilm degradation was also linked to the bactericidal activity of the D-peptide since it induced a strong reduction in the vitality of both detached cells and bacteria encased in the residual biofilm matrix.

The L-native peptide, instead, did not show any ability to reduce biofilm biomass and thickness. However, our results indicate that it was able to kill detached bacteria and slightly impair the ECM architecture. Furthermore, the number of viable bacteria embedded in the ECM was significantly reduced. These data are in line with the observation that some AMPs induce a decrement in the vitality of biofilm-embedded bacteria without reducing biofilm biomass [69]. We might speculate that the lowest capability to degrade the pre-formed biofilm of the L-peptide compared to the D-enantiomer is linked to its high sensitivity to digestion by elastase, produced by bacteria within mature biofilms, which could affect the stability of the peptide, limiting its action in penetration and dissolution of *P. aeruginosa* biofilms.

Taken together, our results indicate that D-Q53 CecB has higher anti-*Pseudomonas* properties compared to the L-peptide in a range of concentrations with low/no in vitro cytotoxicity. The D-form kills planktonic bacteria more efficiently, and it displays better antibiofilm activity, inhibiting its formation and inducing its degradation. Furthermore, it is more resistant to protease digestion, making it a better candidate for biomedical applications. Additionally, the D-peptide is thermostable as it maintained its antimicrobial activity after heat pretreatment for up to 30 min, a favorable characteristic to overcome possible high-temperature treatments required in some biomedical applications for AMPs [70]. Given these capabilities, it will also be interesting to evaluate the possible synergic effects of D-Q53 CecB in combination with conventional antibiotics, known to efficiently work on bacteria in a planktonic state [5,71].

## 4. Materials and Methods

### 4.1. Microbial Strains and Culture Conditions

The following strains were used: *P. aeruginosa* ATCC 27853, ATCC 25668, and PAO1-L (Lausanne subline) [35]. All strains were grown at 37 °C in Luria Bertani (LB) medium or in Luria Bertani Agar (LBA) plates.

### 4.2. Peptides Synthesis

L- and D-CecB Q53 peptides (Figure 1A) were chemically synthesized at the Peptide Synthesis Facility (Dept of Biomedical Sciences, University of Padova, Padua, Italy), employing a solid-phase technique performed on a fully automated peptide synthesizer (Syro II, MultiSynTechGmbh, Witten, Germany). Wang resins preloaded with the first N-α-Fmoc-protected amino acid was employed for stepwise assembly of the entire peptide chain. This assembly was performed according to the Fmoc standard strategy and was based on the use of HATU as the coupling reagent [72,73]. The side-chain protected amino acid building blocks used in the synthesis of L-and D-CecB Q53 peptides were Fmoc-Glu(OtBu)-OH, Fmoc-Gln(Trt)-OH, Fmoc-Asn(Trt)-OH, Fmoc-Ser(tBu)-OH, Fmoc-Lys(Boc)-OH, Fmoc-Arg(Pbf)-OH, Fmoc-Asp(OtBu)-OH, Fmoc-Trp(Boc)-OH. Deprotection of the final peptides and cleavage from the resin was conducted with a mixture of 88% (*v*/*v*) trifluoroacetic acid (TFA) with 5% phenol (*w*/*v*), 5% H_2_O (*v*/*v*) and 2% (*v*/*v*) of triisopropylsylane via shaking at room temperature (RT) for 2.5 h. A step of vacuum filtration permitted to remove the resin from the assembled peptide chains. Then, the peptides were precipitated with cold diethyl ether and transformed into pellets by a centrifugation procedure. Two washes with cold diethyl ether were performed on the precipitated peptides. At the end, purification of the crude peptides was performed through flash chromatography (SP1, Biotage, Uppsala, Sweden) on a Biotage SNAP Ultra C18 12 g cartridge packed with Biotage HP-Sphere C18 25 μm spherical silica. A final step of molecular mass confirmation was performed by mass spectroscopy on a MALDI-TOF/TOF mass spectrometer (ABI 4800, AB Sciex, Framingham, MA, USA). The lyophilized peptides were stored at −20 °C.

### 4.3. Circular Dichroism Analysis

Far-ultraviolet Circular dichroism (CD) spectra of L- and D-Q53 CecB peptides were recorded using a JASCO J-815 spectropolarimeter (Jasco Europe S.r.l., Cremella, Italy) equipped with a Peltier temperature controller and Spectra Manager software 2.0. Suprasil cuvettes with 1.0 mm pathlength were used for all the analyses. Moreover, 10 µM of each peptide was prepared in 30% trifluoroethanol (TFE) in sterile water. Measurements were performed in the 260–195 nm wavelength range with a temperature set at 20 °C. The scanning speed was 100 nm/min, the bandwidth was 2.00 nm, and the data pitch was 0.1 nm.

### 4.4. Enzymatic Digestion of Peptides

L- and D-Q53 CecB peptides, as well as the human LL-37 peptide (Merck, Burlington, MA, USA), were dissolved in 10 mM Tris-HCl, pH 7.5, at a final concentration of 1 mg/mL. Subsequently, 120 μL of each peptide solution were incubated at 37 °C with 20 U of human leukocyte elastase (Merck) or *P. aeruginosa* elastase (Elastin Products Company, Owensville, MO, USA) and 5 μL of 10 mM Tris-HCl, pH 7.5. At given time intervals (30, 60, 120, and 300 min), a volume corresponding to 30 μg of each peptide was diluted in 70 μL of 1% trifluoroacetic acid (TFA) to stop the reaction. Peptide suspensions,v without any enzyme (undigested controls), were processed in parallel. Each solution was analyzed by RP-HPLC, using a Phenomenex LC C18 analytical column (300 Å, 5 mm, 250 × 4.6 mm; Phenomenex, Torrance, CA, USA) and a Widepore C18 security guard cartridge (4 × 3.0 mm; Phenomenex, Torrance, CA, USA), in a 30 min-linear Acetonitrile (ACN) gradient (from 0% to 40% for L- and D-Q53 CecB peptides; from 20% to 60% for LL-37). A Waters Alliance 2695 Separations Module connected to a 996 PDA (Waters, Milford, MA, USA) was employed for RP-HPLC.

### 4.5. MIC and MBC Tests

The antibacterial activities of L- and D-Q53 CecB were evaluated by determining (i) the MIC and (ii) the MBC. Tobramycin (Merck) was used as an antibiotic control. (i) An overnight (ON) bacterial culture was diluted to 1 × 10^6^ cells/mL in LB medium. 50 µL/well were seeded in sterile polypropylene 96-well plates (Corning, Corning, NY, USA) and added with 50 µL LB medium, containing serial dilutions of each compound (final concentrations of L- and D-Q53 CecB peptides from 1.1 to 35.2 µM; final concentrations of tobramycin from 0.26 to 17 μM; each condition in triplicate). Microbial suspensions in the growing medium (positive control) and wells with only the medium (negative controls) were processed in parallel. Plates were incubated 24 h at 37 °C with agitation (300 rpm), and the absorbance was measured at 600 nm in a microplate reader. The MIC was considered as the lowest concentration determining a delta optical density at 600 nm (ΔOD_600_) equal to 0 ± 0.05. The ΔOD_600_ was determined by subtracting the OD_600_ recorded at time 0 from that obtained after 24 h. Each experiment was performed at least three times. (ii) To establish the MBC, dilutions of each compound (final concentrations of L- and D-Q53 CecB peptides from 2.2 to 22 μM; final concentrations of tobramycin from 2.14 to 17 μM) were incubated in duplicate with 100 μL of microbial culture (5 × 10^5^ cells/mL) for 24 h at 37 °C. For each sample, 100 μL of 1:10, 1:100, and 1:1000 dilutions were plated on LBA plates, and colonies were counted after 24 h at 37 °C. The MBC was considered the peptide concentration able to inhibit 99.9% of bacterial growth. Each experiment was performed at least twice.

### 4.6. Resistance of L- and D-Q53 CecB to Salts, Serum, and Heat Treatments

Effects of (i) NaCl, (ii) CaCl_2_, (iii) MgCl_2_, and (iv) inactivated FBS on L- and D-Q53 CecB activity were investigated by performing MIC assays on bacterial cultures grown in LB medium containing: (i) 0, 100, 200, 300, 400 mM NaCl; (ii) 0, 1, 2 mM CaCl_2_; (iii) 0, 0.5, 1 mM MgCl_2_; (iv) inactivated FBS (Thermo Fisher Scientific, Waltham, MA, USA) at a final concentration of 0, 10, and 20%. To evaluate the peptide stability at high temperatures, 300 μM peptide solutions were treated at 100 °C for 0, 5, 10, and 30 min. MICs were determined as described above.

### 4.7. Inhibition of Biofilm Formation

An ON bacterial culture was diluted to 2 × 10^6^ cells/mL in an LB medium. 50 µL/well were seeded in a sterile non-treated polystyrene 96-well plate (Corning) and added with 50 µL LB medium containing serial dilutions of each compound (final concentrations of L- and D-Q53 CecB peptides: from 0.034 to 17.6 µM; final concentrations of tobramycin: 0.27 and 0.53 μM, and from 2.14 to 17 μM). Each condition was tested in three-six replicates. Wells containing untreated bacteria (negative controls) or LB medium only (blank) were followed in parallel. Each plate was incubated for 24 h at 37 °C without agitation. After washing twice, samples were stained with a 0.1% crystal violet solution for 20 min, washed three times, and incubated in 30% acetic acid for 20 min, as in [74]. Plates were read at 595 nm using a microplate reader. For each sample, the biofilm biomass percentage was calculated relative to that of the untreated control showing the highest OD value, set equal to 100%. Each experiment was performed at least twice.

### 4.8. Biofilm Degradation

One hundred µL of bacteria (1 × 10^6^ cells/mL), derived from a fresh ON culture, were seeded in a sterile non-treated polystyrene 96-well plate (Corning) and incubated for 24 h at 37 °C without agitation. After washing, 24 h old biofilms were incubated with 100 µL of LB medium, containing serial dilutions of each compound (from 1.1 to 17.6 µM for both L- and D-Q53 CecB peptides; 0.55 µM for D-Q53 CecB only; from 2.14 to 17 μM for tobramycin; four replicates per condition) for 1 h at 37 °C without agitation. Evaluation of biofilm biomass was performed using a crystal violet assay as described above (Section 4.8). Experiments were performed two-four times. After 2.2 µM L- and D-Q53 CecB treatments, the number of viable bacteria in the supernatant was also evaluated. 20 µL of each supernatant were diluted as necessary, and the CFUs were counted (four experiments, one replicate each).

### 4.9. In Vitro Interactions between DNA and CecB Peptides

One hundred ng of linearized DNA (pIVEX 2.3d plasmid) were incubated with increasing concentrations of the L- or D-peptides (0, 100, 150, 175, 200, 250, 275, 300, 350, 400, 450, 500 ng) at RT for 1 h. Samples were run into a 1% agarose gel in TAE buffer at 50 V and visualized in a Gel Doc X3+ (Biorad, Hercules, CA, USA). DNA band intensities were measured using the Image Lab software 6.1 (Biorad, Hercules, CA, USA). The band intensity reduction was calculated as the ratio between the band intensity in the presence of peptide over that of the untreated DNA. To compare the DNA binding activities of L- and D- peptides, for each peptide amount, we determined the ratio between the band intensity reduction with D-Q53 CecB and that with L-Q53 CecB. Three experiments have been performed.

### 4.10. P. aeruginosa Growth Curves with sub-MIC Concentrations of L- and D-Q53 CecB Peptides

Bacteria (5 × 10^5^ cells/mL), derived from a fresh ON culture, were incubated in sterile polypropylene 96-well plates (Corning) with serial dilutions of the peptides (final concentrations from 0.275 to 2.2 µM, each condition in triplicate), as for the MIC assay (Section 4.4). Microbial suspensions in LB medium (positive controls) and wells with LB medium only (negative controls) were followed in parallel. Plates were incubated at 37 °C with agitation (300 rpm), and the absorbance was measured every 2 h for 14 h at 600 nm. For each time point, a ΔOD_600_ was determined by subtracting the OD_600_ recorded at time 0 from those obtained at the different time-points. To establish the peptide concentration not inhibiting bacterial growth, for each treatment, the Area Under the Curve (AUC) was calculated and compared to that of untreated controls.

### 4.11. Motility Assay

The motility assay was performed as in [36], with minor modifications. Bacteria were grown in an LB medium containing sub-MIC concentrations of the L- and D-peptides (1.1 and 0.55 µM for L- and D-Q53 CecB, respectively) as described above (Section 4.11) and inoculated in plates (60 × 15 mm), containing LB medium with variable concentrations of agar (0.3, 0.5, and 1%, to detect swimming, swarming, and twitching, respectively), supplemented with 1.1 µM L-Q53 CecB or 0.55 µM D-Q53 CecB. Inocula were performed with a sterile toothpick (i) within the medium to detect swimming, (ii) on the top of the medium to detect swarming, and (iii) on the bottom of the plate to detect twitching. Bacteria grown and plated in the absence of any peptide were processed as negative controls. Plates were incubated at 30 °C (for swimming and swarming) or 37 °C (for twitching) and monitored for 24–48 h. Plate images were taken with a digital camera. Post-acquisition analyses were performed with Fiji, an open-source image-processing package based on ImageJ (http://fiji.sc/wiki/index.php, downloaded on 4 April 2022). Swimming, swarming, and twitching data were obtained by calculating the area of the growing zone over the total area of each plate. Values were expressed as percentages. Each type of motility was evaluated in at least four independent experiments, each with one replica per condition.

### 4.12. EPS Determination

Experiments were performed in LB medium supplemented 2% glucose to enhance the EPS production, as in [46]. Five mL of bacterial cultures (1 × 10^6^ cells/mL) were added to the same volume of the peptide at a dilution not-inhibiting the bacterial growth (final concentrations 1.1 and 0.55 µM for L- and D-Q53 CecB, respectively, as in Section 4.11) and incubated at 37 °C for 20 h with agitation (200 rpm). 10 mL of bacterial culture (5 × 10^5^ cells/mL) without any peptide were grown in parallel. Samples were incubated at 37 °C for 20 h with agitation. Glucan levels were determined as in [46,75] with some modifications. For each sample, 5 mL were centrifuged at 5000× *g* for 10 min, and supernatants were collected in clean tubes; pellets were washed twice in 2.5 mL autoclaved water and centrifuged at 5000× *g* for 10 min, collecting supernatants again. Three volumes of chilled absolute ethanol were added to the collected supernatants and placed at 4 °C ON to precipitate polysaccharides. After centrifugation at 10,000× *g* for 10 min, pellets were washed with chilled ethanol 70% and resuspended in 5 mL 0.1 M NaOH. Two mL of 5% aqueous solution of phenol and 5 mL of concentrated sulphuric acid were added. Mixtures were incubated 10 min at RT in the dark, vortexed 30 sec, placed in a water bath at 25 °C 20 min and read at 490 nm. The experiment was performed four times, each condition in duplicate.

### 4.13. Real-Time qPCR

Bacteria were grown in an LB medium containing sub-MIC concentrations of the L- and D-peptides (1.1 and 0.55 µM for L- and D-Q53 CecB, respectively, Section 4.11). Bacteria were collected after centrifugation and stored in Trizol Reagent (Ambion, Waltham, MA, USA) at −80 °C, until use. Total RNA was extracted using the Direct-zol RNA MiniPrep kit (Zymo Research, Irvine, CA, USA). The complementary DNA (cDNA) was synthesized using the Maxima™ H Minus cDNA Synthesis Master Mix with dsDNase (Molecular biology, Thermo Scientific). Each qPCR reaction was performed in triplicate, using the GoTaq qPCR Master Mix (Promega, Madison, WI, USA) and a CFX96 Real-Time PCR Detection System (Bio-Rad, Hercules, CA, USA), following the manufacturer’s instructions. The amplification conditions were 95 °C 2 min, (15 s at 95 °C, 1 min at the appropriate annealing temperature Ta, as in Table 5) ×40 cycles. The *rplU* gene, coding the 50 S ribosomal protein, was used to normalize qPCR data as in [36]. Primer sequences are reported in Table 5. Fold changes were calculated by the 2^−ΔΔCT^ analysis method.

### 4.14. Confocal Fluorescence Microscopy

Bacteria cells (1 × 10^6^ cells/mL) were grown in Permanox four-wells chamber slide systems (1.8 cm^2^/well; Nunc^®^ Lab-Tek^®^ Chamber Slide™ system) at 37 °C for 24 h without agitation to allow biofilm formation. After washing, biofilms were treated with 2.2 μM L- or D-Q53 CecB peptide for 1 h at 37 °C without agitation. Negative controls, treated with LB only, were followed in parallel.

To visualize biofilm-embedded cells, EPS, and protein components of the biofilm matrix, samples were stained in the dark, with 12. 5 μM SYTO™ 9 (Thermo Fisher Scientific), for 30 min to detect bacteria (green), with 1:1 Calcofluor White (Merck) for 1 min to detect EPS (blue), and with SYPRO™ Ruby Biofilm Matrix Stain (Thermo Fisher Scientific) for 30 min to visualize proteins (red). After each staining, samples were washed twice with PBS. After washing, samples were fixed in 4% Paraformaldehyde, mounted in VECTASHIELD^®^ mounting medium (Vector Laboratories, Newark, CA, USA), and observed using a Zeyss LSM 900 Airyscan 2 confocal microscope and a ×63 oil immersion objective. Optical sections (Z-series) were taken at 0.2 μm intervals. Experiments were performed four times (one-two replicates per condition).

To visualize live/dead bacteria, samples were stained with 12. 5 μM SYTO™ 9 (Thermo Fisher Scientific) (green, total cells) and 70 μM Propidium Iodide (Merck) (red, dead cells) for 30 min. After washing with PBS, samples were mounted in VECTASHIELD^®^ mounting medium (Vector Laboratories) and observed using a Leica TCS SP5 II confocal microscope, using a ×63 oil immersion objective and a digital magnification of ×3. Optical sections (Z-series) were taken at 0.13 μm intervals. For each condition, experiments were repeated at least twice.

To detect bacteria (green), excitation and emission wavelengths were 488 and 500–530 nm, respectively. EPS was visualized using a 405 nm wavelength for excitation and 413–480 nm for emission (blue); to detect proteins or Propidium Iodide (red), a 543 nm wavelength for excitation and 590–610 nm for emission were used. Post-acquisition analyses were performed with Fiji (http://fiji.sc/wiki/index.php, downloaded on 4 April 2022). Cross-section views (x-z and y-z planes were obtained with the “Orthogonal View” plugin. To evaluate live and dead cells, images of single 0.13 μm slices taken in the upper part of the biofilm (i.e., in contact with the peptide during treatment) were processed using the “Counting Objects” plugin, and the same threshold level for red and green colors.

### 4.15. Hemolytic Assay

The peptide hemolytic activity was evaluated against human red blood cells (hRBCs) using the Haemolysis Assay Kit (HaemoScan, Groningen, The Netherlands), following the manufacturer’s instructions. 180 μL of hRBC solution were incubated 1 h at 37 °C with 20 μL of serially diluted L- and D-CecB Q53 peptides (final concentrations of 5, 10, 25, 50, 100, 200 µM) in triplicate. As hemolysis positive and negative controls, hRBC solutions incubated, respectively, with 0.2% Triton X and PBS, were followed in parallel. After centrifugation 10 min at 10,000× *g*, each supernatant was diluted 1:10 in the Assay Buffer and read at 450 nm with a microplate reader.

Hemolysis was evaluated according to the following equation:

$$\mathrm{Hemolysis}\;(\%)=\frac{{OD}_{CecB}-{OD}_{0}}{{OD}_{Ctrl}-{OD}_{0}}\times 100,$$

where OD_CecB_ is the OD in the presence of the peptide, OD_Ctrl_ is the OD of treated controls (0.2% Triton X), and OD_0_ is the background absorbance. The experiment was performed at least twice.

### 4.16. Cytotoxicity Assay

The peptide cytotoxicity against human cells was evaluated using CCD-34Lu-lung fibroblasts and A549-lung adenocarcinoma cells. Moreover, 6 × 10^3^ cells in DMEM medium added with 10% FBS were seeded in 96-well sterile plates. After 24 h, cells were incubated with increasing concentrations of peptide (final concentrations of 5, 10, 25, 50, and 100 µM, with an additional concentration of 200 µM in CCD-34Lu) for 24 h at 37 °C (three replicates per condition). Cell viability was measured by removing the exhausted medium, adding 100 µL of DMEM and 20 µL of the MTS solution (Promega). Plates were incubated at 37 °C for 20 min until the complete conversion of the compound. The absorbance was measured at 492 nm using a microplate reader. Cell viability was calculated using the formula:

$$\mathrm{Viability}\;(\%)=\frac{{OD}_{CecB}-{OD}_{0}}{{OD}_{Ctrl}-{OD}_{0}}\times 100,$$

where OD_CecB_ is the OD in the presence of the peptide, OD_Ctrl_ is the OD of cells without the peptide, and OD_0_ is the background absorbance (wells with only medium and MTS compound). The experiment was performed at least twice.

### 4.17. Statistical Analysis

All data were checked for normality using Kolmogorov–Smirnov and Shapiro–Wilk tests. Those approximating a normal distribution were parametrically analyzed with a one-way ANOVA, followed by Dunnett’s or Tukey’s post hoc tests for multiple comparison evaluations. qPCR and red/green cells ratio data did not approximate normal distributions; thus, they were analyzed using a Kruskal–Wallis test, followed by Dunn’s post hoc test. Peptides’ hemolytic activity data were compared using a mixed-effects model followed by a post hoc Sidak test. Analyses were performed using GraphPad Prism 10.0.0.

## Figures and Tables

**Table 1:** MIC and MBC values of L-, D-Q53 CecB peptides, and tobramycin against *P. aeruginosa* ATCC 25668, ATCC 27853, and PAO1-L strains.

Compound	MIC (μM)	MBC (μM)
D-Q53 CecB	2.20	4.40 (2.20) *
L-Q53 CecB	2.20	11 (4.40) *
Tobramycin	2.14	4.29 (8.56) *

* Data between parentheses indicate MBC values obtained against the PAO1-L strain.

**Table 2:** MIC values of L- and D-Q53 CecB peptides against *P. aeruginosa* ATCC 27853 strain evaluated at different concentrations of NaCl, CaCl_2_, MgCl_2,_ FBS, and after heat treatments.

Condition	D-Q53 CecB MIC (μM)	L-Q53 CecB MIC (μM)
NaCl 100 mM	2.2	2.2
NaCl 200 mM	4.4	2.2
NaCl 300 mM	8.0	8.0
NaCl 400 mM	>8.0	>8.0
CaCl_2_ 1 mM	4.4	8.0
CaCl_2_ 2 mM	15	15
MgCl_2_ 1 mM	4.4	2.2
MgCl_2_ 0.5 mM	2.2	2.2
FBS 20%	8.0	5.5
FBS 10%	4.4	4.4
100 °C 5 min	2.2	2.2
100 °C 10 min	2.2	2.2
100 °C 30 min	2.2	2.2

**Table 3:** Detachment of living bacterial cells from 24 h-old biofilms after 1 h treatment with 1 MIC of L- and D-Q53 CecB.

*P. aeruginosa* Strain	Control (CFUs/mL) Mean ± SEM	D-Q53 CecB (CFUs/mL) Mean ± SEM	L-Q53 CecB (CFUs/mL) Mean ± SEM
ATCC 27853	3.06 ± 2.07 × 10^14^	1.07 ± 1.06 × 10^10^	7.03 ± 4.10 × 10^8^
ATCC 25668	2.67 ± 2.67 × 10^15^	6.70 ± 6.6 × 10^8^	2.89 ± 2.38 × 10^5^

**Table 4:** MHC, IC_50_, and SI for L- and D-Q53 CecB peptides.

Peptide	MHC (μM)	CCD-34 Lu		A549	
		IC_50_ (μM)	SI	IC_50_ (μM)	SI
D-Q53 CecB	100	72.59 ± 2.81	16.47	68.02 ± 3.71	15.45
L-Q53 CecB	>200	>200	>18.18	>100	>9.09

MHC is the minimum hemolytic concentration causing 5% hemolysis of hRBCs. IC_50_ is reported as mean ± SEM. SI is equal to the mean value of IC_50_ divided by MBC obtained against *P. aeruginosa* ATCC 27853 and ATCC 25668 strains; “>” is reported when the highest tested concentration did not show any measurable toxic effects.

**Table 5:** Primers used for RT-qPCR.

Gene	Primer	Sequence (5′ -> 3′)	Ta (°C)	Ref.
*lasI*	lasI-F	GGCGCGAAGAGTTCGATAAA	57	[36]
	lasI-R	CCATCTCGTCGATGACACTAAC		
*rpoS*	rpoS-F	CGGCGAGTTGGTCATCATCAAACA	63	[36]
	rpoS-R	ATCGATTGCCCTACCTTGACCTGTC		
*fimX*	fimX-F	CCTGGCCTATATCCATCTCAAC	57	[36]
	fimX-R	ACTGTTCACGCATCAGTCC		
*pelA*	pelA-F	CCTTCAGCCATCCGTTCTTCT	59	[18]
	pelA-R	TCGCGTACGAAGTCGACCTT		
*pslA*	pslA-F	AAGATCAAGAAACGCGTGGAAT	57	[18]
	pslA-R	TGTAGAGGTCGAACCACACCG		
*algD*	algD-F	GCGACCTGGACCTGGGCT	58	[76]
	algD-R	TCCTCGATCAGCGGGATC		
*rplU*	rplU-F	CGCAGTGATTGTTACCGGTG	59	[36]
	rplU-R	AGGCCTGAATGCCGGTGATC		

## Data Availability

In Supplementary Materials, Figures S1 and S2 reported the RP-HPLC profiles of L-Q53 CecB, D-Q53 CecB, and LL-37 peptides after 0, 60, and 300 min of incubation with the human neutrophil and *P. aeruginosa* elastases, respectively. Figures S5–S7 reported the gel images obtained when evaluating the interaction between increasing amounts of L- or D-Q53 CecB peptides and 100 ng of linearized DNA in the first, second, and third experiments, respectively.

## Supplementary Materials

The supporting information can be downloaded at: https://www.mdpi.com/article/10.3390/ijms241512496/s1.
