# Supplementary material for: Fighting Pseudomonas aeruginosa Infections: Antibacterial and Antibiofilm Activity of D-Q53 CecB, a Synthetic Analog of a Silkworm Natural Cecropin B Variant
Source: Int J Mol Sci. 2023 Aug 6;24(15):12496. doi: 10.3390/ijms241512496 (PMC10419416; doi:10.3390/ijms241512496)
Supplement: Supplementary file 1 [file ijms-24-12496-s001.zip › ijms-2548866_original gels-Figures S5-S7.pdf]

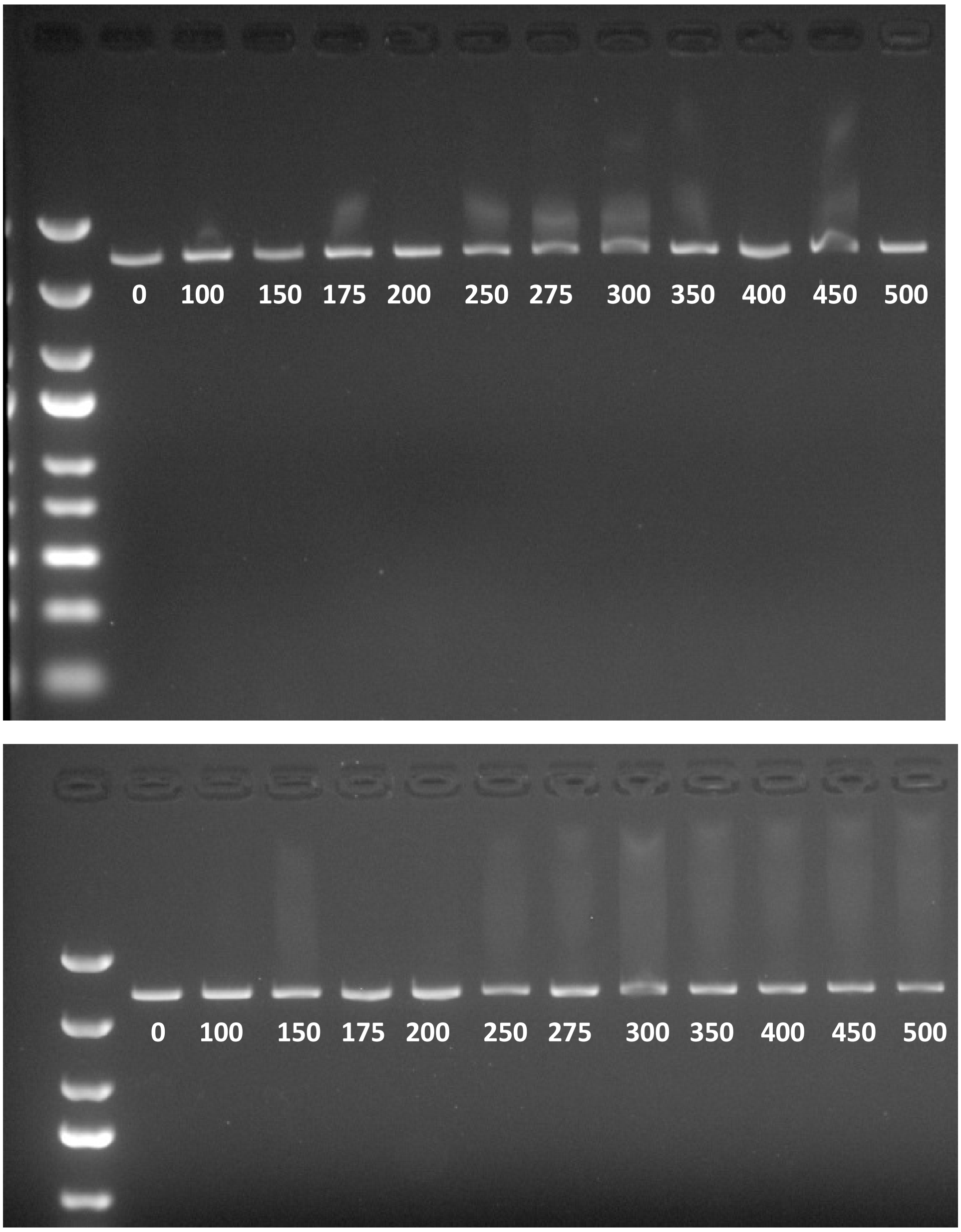

Figure S5. Interaction between increasing amounts of L- or D-Q53 CecB peptides and 100 ng of linearized DNA. Gel images of the first experiment; Amounts of peptides (ng) are reported in white.

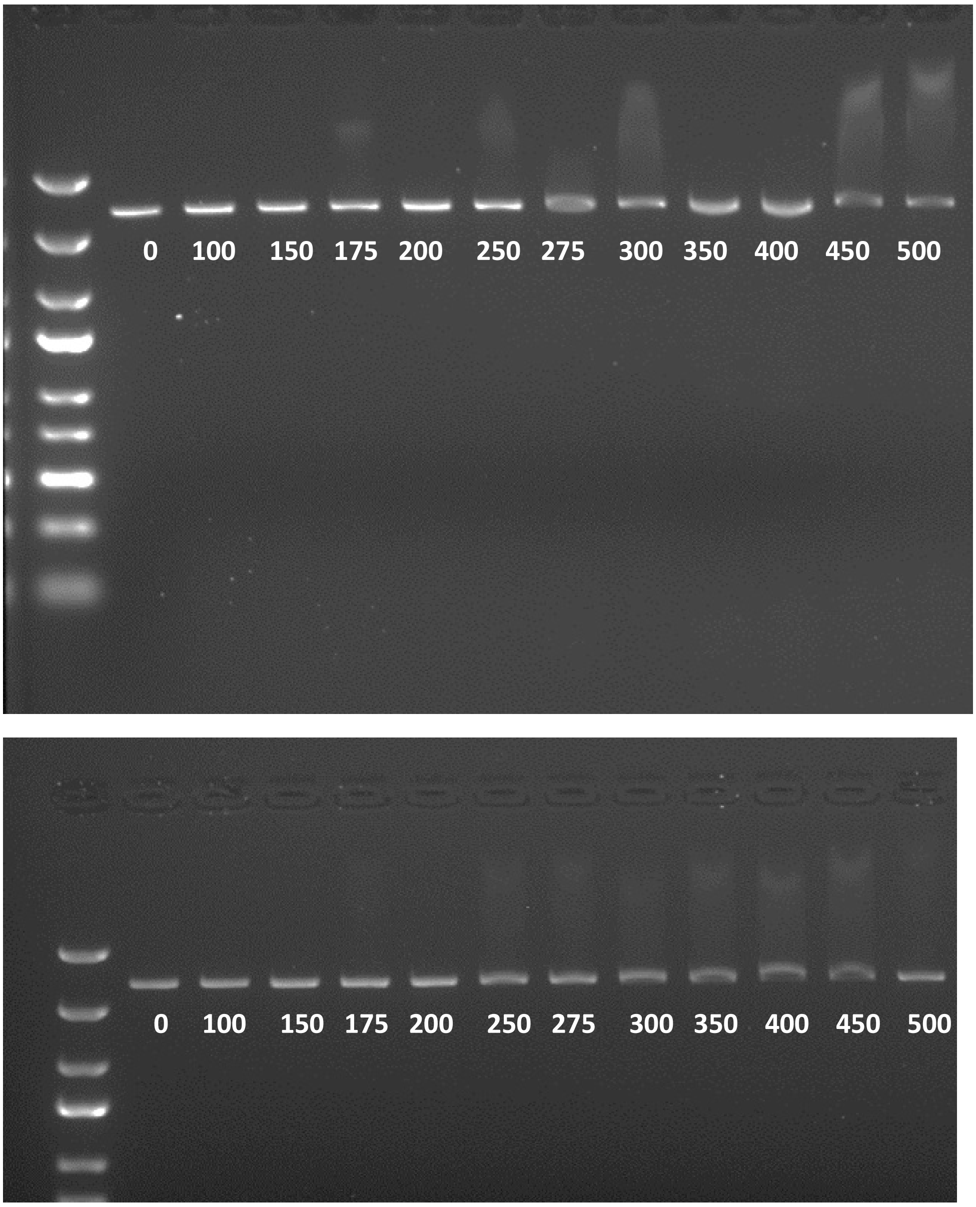

L-Q53 CecB

D-Q53 CecB

Figure S6. Interaction between increasing amounts of L- or D-Q53 CecB peptides and 100 ng of linearized DNA. Gel images of the second experiment; Amounts of peptides (ng) are reported in white.

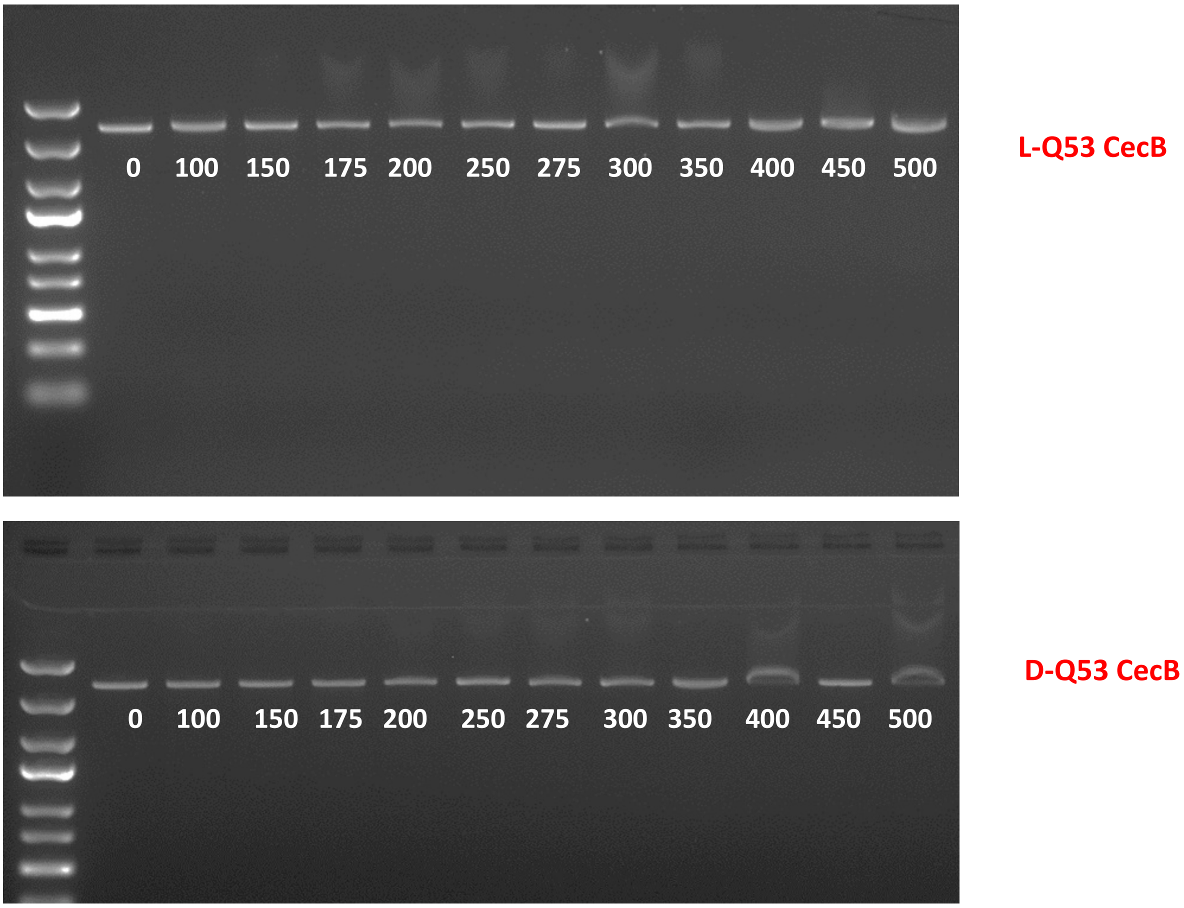

Figure S7. Interaction between increasing amounts of L- or D-Q53 CecB peptides and 100 ng of linearized DNA. Gel images of the third experiment; Amounts of peptides (ng) are reported in white.
