# Supplementary material for: Fighting Pseudomonas aeruginosa Infections: Antibacterial and Antibiofilm Activity of D-Q53 CecB, a Synthetic Analog of a Silkworm Natural Cecropin B Variant
Source: Int J Mol Sci. 2023 Aug 6;24(15):12496. doi: 10.3390/ijms241512496 (PMC10419416; doi:10.3390/ijms241512496)
Supplement: Supplementary file 1 [file ijms-24-12496-s001.zip › ijms-2548866_Supplementary Materials-Figures S1-S4.pdf]

Supplementary Materials

Fighting *Pseudomonas aeruginosa* Infections: Antibacterial and Antibiofilm Activity of D-Q53 CecB, a Synthetic Analog of a Silkworm Natural Cecropin B Variant

Irene Varponi [1], Stefania Ferro [2], Luca Menilli [1], Alessandro Grapputo [1,3], Francesca Moret [1], Francesca Mastrotto [4], Oriano Marin [2] and Federica Sandrelli [1,*]

[1] Department of Biology, University of Padova, via U. Bassi 58/B, 35131 Padova, Italy
[2] Department of Biomedical Sciences, University of Padova, via U. Bassi 58/B, 35131 Padova, Italy
[3] National Biodiversity Future Centre, Piazza Marina 61, 90133 Palermo, Italy
[4] Department of Pharmaceutical and Pharmacological Sciences, University of Padova, via F. Marzolo 5, 35131 Padova, Italy

* Correspondence: federica.sandrelli@unipd.it; Tel.: +39-049-8276216

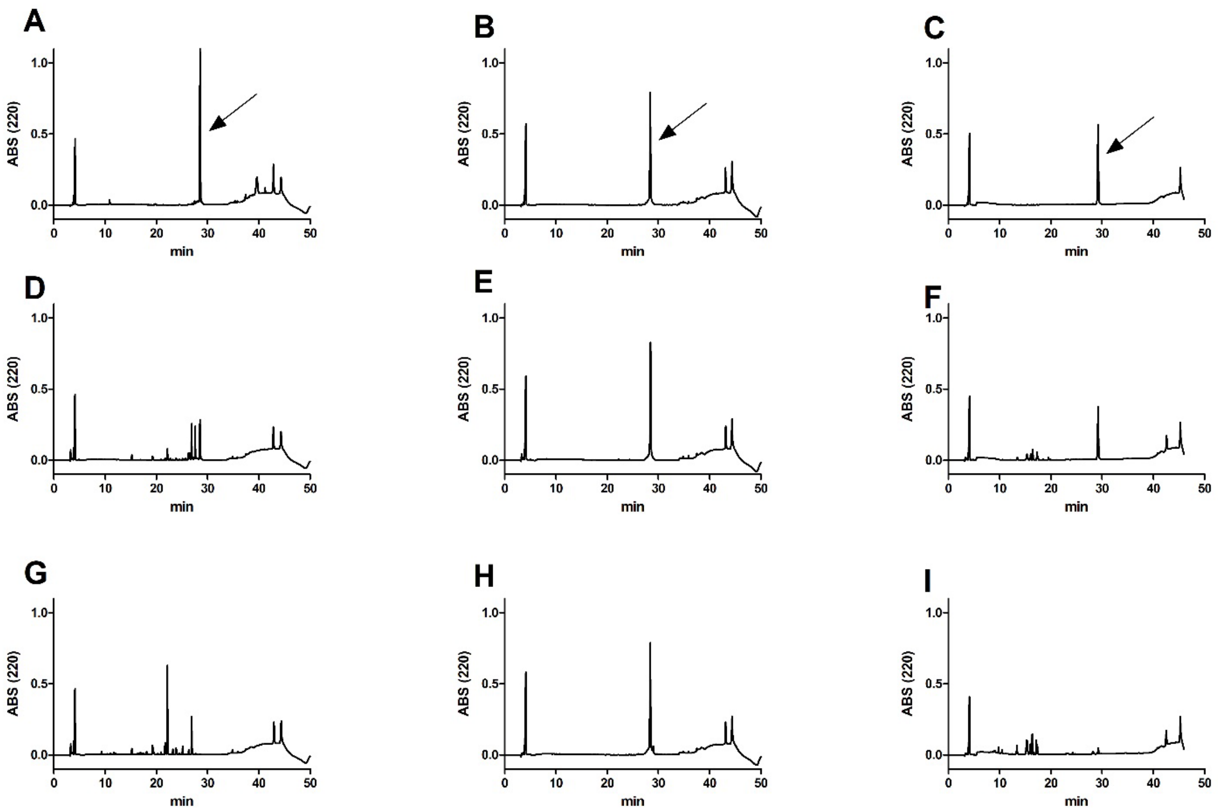

Supplementary Figure S1. Proteolytic activity of human neutrophil elastase on L-Q53 CecB, D-Q53 CecB and LL-37 peptides. RP-HPLC profiles of peptides after 0 min (**A, B, C**), 60 min (**D, E, F**) and 300 min (**G, E, H**) of incubation with the proteolytic enzyme. In **A, B, and C** arrows indicate the peaks of the intact peptides at their respective retention time.

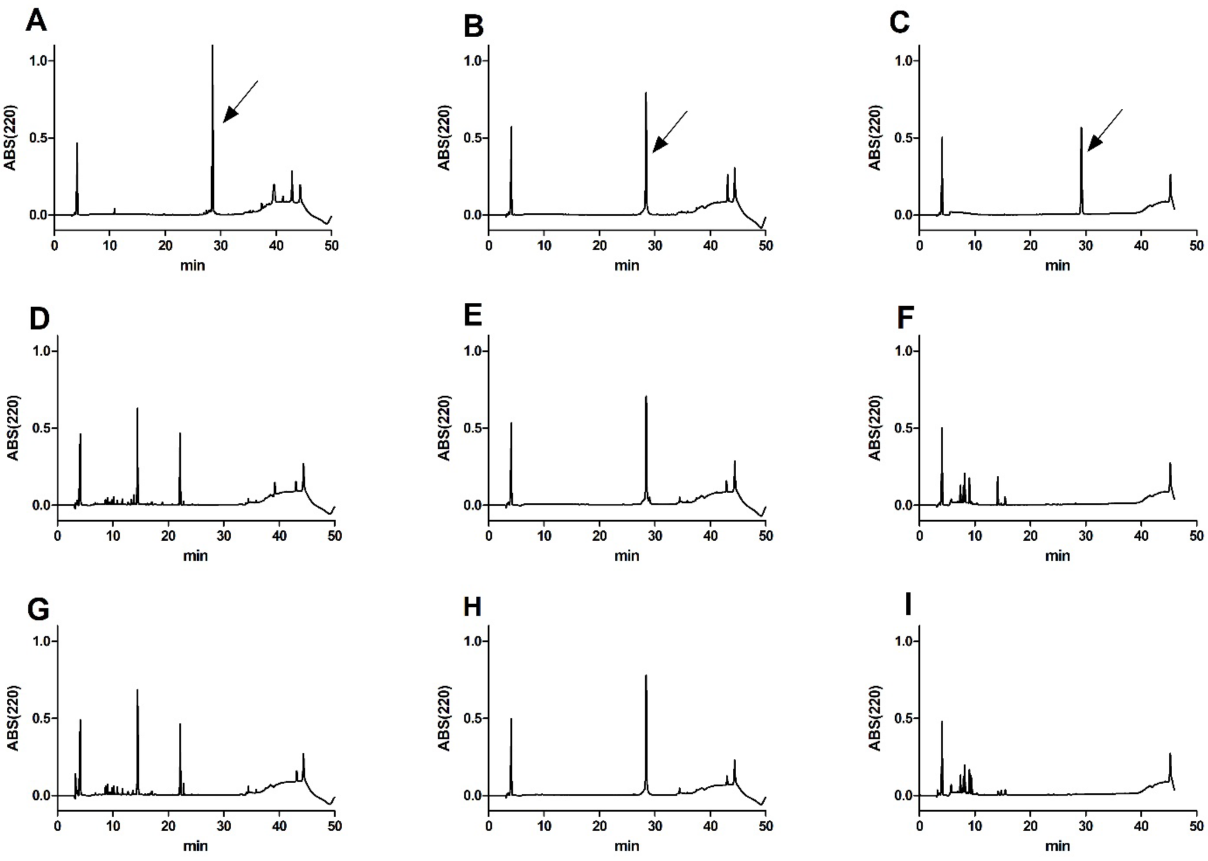

Supplementary Figure S2. Proteolytic activity of *P. aeruginosa* elastase on L-Q53 CecB, D-Q53 CecB and LL-37 peptides. RP-HPLC profiles of peptides after 0 min (**A, B, C**), 60 min (**D, E, F**) and 300 min (**G, E, H**) of incubation with the proteolytic enzyme. In **A, B, and C** arrows indicate the peaks of the intact peptides at their respective retention time.

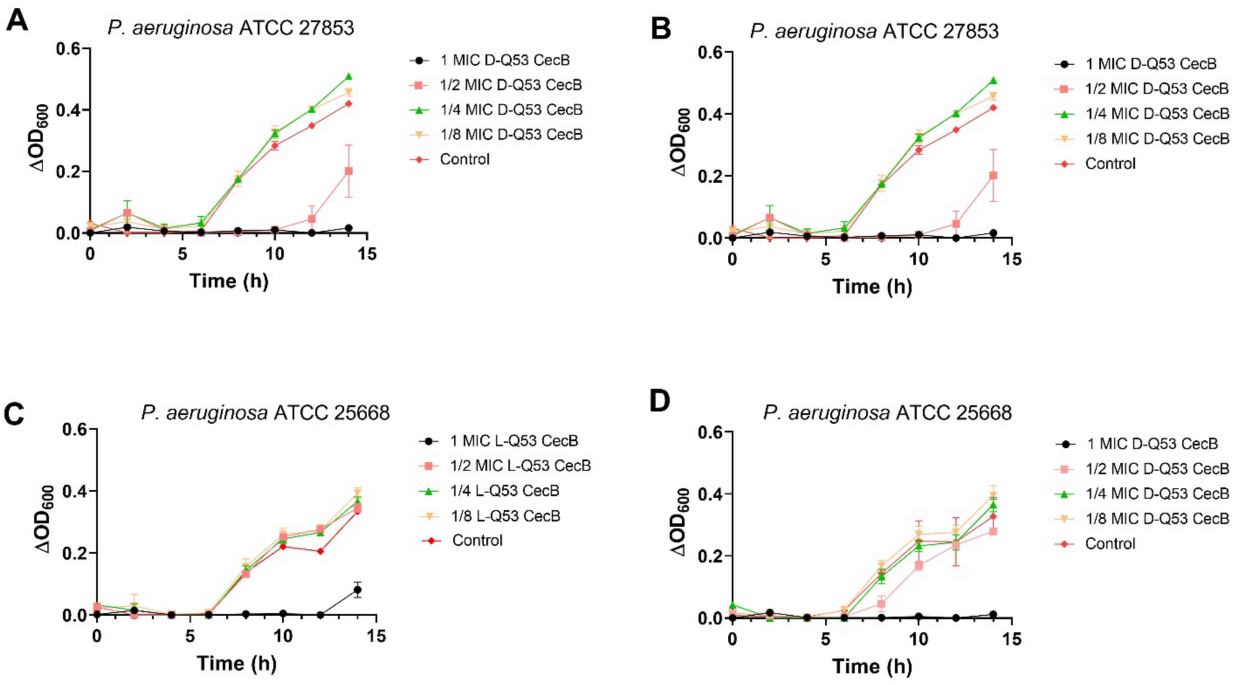

Supplementary Figure S3. Growth curves of *P. aeruginosa* treated with of L-Q53 CecB and D-Q53 CecB peptides at sub-MIC concentrations. ATCC 27853 strain treated with (**A**) L-Q53 CecB and (**B**) D-Q53 CecB. ATCC 25668 strain treated with (**C**) L-Q53 CecB and (**D**) D-Q53 CecB. Bacteria treated with 1 MIC L- or D- Q53 CecB peptides and untreated bacteria represent positive and negative controls, respectively. One-way ANOVA followed by *post-hoc* Dunnett's tests revealed no significant differences in (**A** and **C**) for the comparison between 1/2 MIC (1.1 μM) L-Q53 CecB treated samples and negative controls, and in (**B** and **D**) for the comparison between 1/4 MIC (0.55 μM) D-Q53 CecB treated samples and negative controls [ATCC 27853: (**A**) $F_{4,10} = 257.8$, $p < 0.0001$; 1/2 MIC L-Q53 CecB vs negative controls*:* Dunnett's test $p > 0.05$, ns; (**B**) $F_{4,10} = 256.9$, $p < 0.0001$; 1/4 MIC D-Q53 CecB vs negative controls*:* Dunnett's test $p > 0.05$, ns; ATCC 25668: (**C**) $F_{4,10} = 171.6$, $p < 0.0001$; 1/2 MIC L-Q53 CecB vs negative controls*:* Dunnett's test $p > 0.05$, ns; (**D**) $F_{4,10} = 43.58$, $p < 0.0001$; 1/4 MIC D-Q53 CecB vs negative controls*:* Dunnett's test $p > 0.05$, ns].

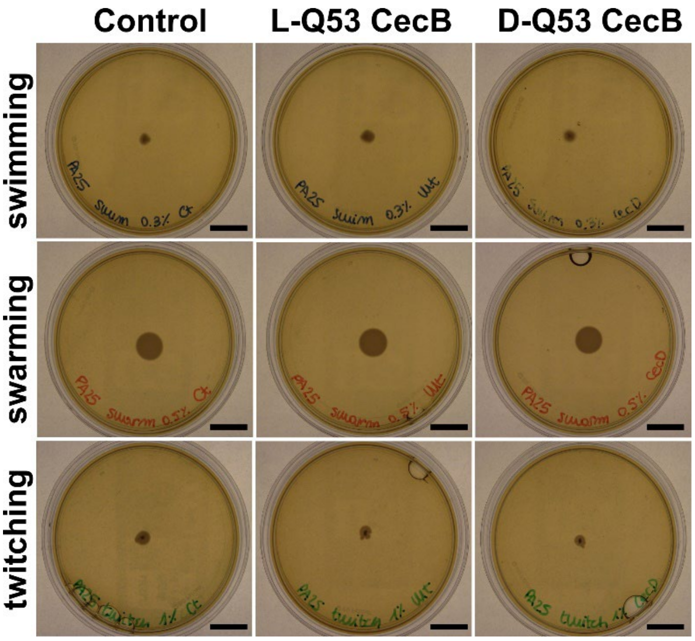

Supplementary Figure S4. Representative images of swimming, swarming and twitching motilities of *P. aeruginosa* ATCC 25668 strain in the presence of L-Q53 CecB (L-Q53), D-Q53 CecB (D-Q53), and untreated controls, after 48 h at 30 °C (swimming and swarming) and 37 °C (twitching). Bars represent 1 cm.
